# In Vitro Structural and Functional Studies of a Novel Cupredoxin, FtrB, from *Brucella abortus* 2308

**DOI:** 10.1021/acsomega.5c00690

**Published:** 2025-03-22

**Authors:** Alexa Kerkan, Kai Hart, Daniel W. Martin, Jason Pajski, Bridget Aidoo, Brandon L. Garcia, Sourav Roy, Saumya Dasgupta, Shabnam Hematian, Andrea Santisteban-Veiga, Nicholas Joseph Schaaf, Sambuddha Banerjee

**Affiliations:** † Department of Chemistry, East Carolina University, Science and Technology Building, Room 409, Greenville, North Carolina 27858, United States; ‡ Department of Microbiology and Immunology, Brody School of Medicine, East Carolina University, Greenville, North Carolina 27852, United States; § Department of Biochemistry and Molecular Biophysics, Kansas State University, Manhattan, Kansas 66506, United States; ∥ Department of Chemistry, Amity University Kolkata, Kolkata, WB 700134, India; ⊥ Department of Chemistry and Biochemistry, University of North Carolina at Greensboro, Greensboro, North Carolina 27402, United States; # AFFINImeter Scientific & Development Team, Software 4 Science Developments, Avenida do Mestre Mateo, 2, 15706 Santiago de Compostela, A Coruña, Spain; ∇ University of Santiago de Compostela, Santiago de Compostela 15782, Spain; ○ University of Santiago de Compostela, Rúa de José María Suárez Núñez, s/n, 15782 Santiago de Compostela, A Coruña, Spain; ◆ Colloids and Polymers Physics Group, Institute of Materials (iMATUS), Department of Applied Physics, University of Santiago de Compostela, 15782 Santiago de Compostela, A Coruña, Spain

## Abstract

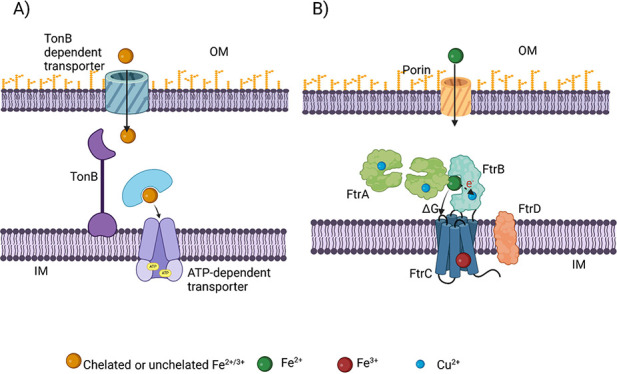

FtrABCD is a four-component iron transporter found in several Gram-negative bacteria. Previous data confirm that FtrABCD can only utilize Fe^2+^ and the inner membrane permease, FtrC, from this system, like its eukaryotic homologue, Ftr1p, is predicted to utilize the free energy released during Fe^2+^ oxidation for the transport. Periplasmic FtrB from this system is coancestral with known copper oxidases, and the conserved D118 and H121 are predicted to bind to Cu^2+^, forming an active enzyme. In this work, we report structural data for recombinant wild-type and D118A and H121A mutants from *Brucella abortus* 2308 which confirm a β-sheet-rich structure which is distinct from known cupredoxins. Calorimetric studies on the wild-type protein show μM affinities for Cu^2+^ and an Fe^2+^ mimic (Mn^2+^), which facilitate the formation of the active enzyme and the enzyme–substrate complex, respectively. In contrast, the D118A mutant failed to bind Cu^2+^. Finally, the electrochemical data reported here revealed biologically accessible reduction potentials for the Cu^2+^ ion in the active enzyme which also showed a pseudozero-order rate of Fe^2+^ oxidation at pH 6.5 and could oxidize Fe^2+^ 3.5-times faster than its rate of autoxidation. Taken together, this report provides experimental data that support structural and functional predictions of FtrB under in vitro conditions.

## Introduction

**Figure 1:**
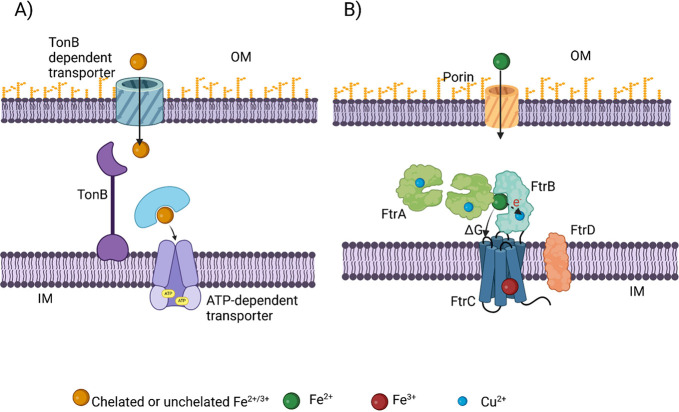
Schematic representations of (A) iron translocation through characterized iron transporters using ATP/GTP-derived energy. The iron-containing cargo (chelated or unchelated) penetrates the outer membrane of Gram-negative bacteria using TonB-dependent β-barrel transporters. In the periplasm, it is trafficked to the dedicated ATP-dependent transporter using a periplasmic binding protein;^1,2^ (B) the proposed functional model for FtrABCD-mediated Fe^2+^ utilization in *Brucella* and *Bordetella* spp.^3−5^ It is proposed that the free energy of Fe^2+^ oxidation achieved by the ferrous oxidase Cu^2+^–FtrB is utilized by FtrC, the inner membrane permease, for Fe^3+^ translocation across the inner membrane.

Iron is a redox active first row transition metal and plays essential roles in the life processes of most living organisms.^1^ However, the predominant oxidation state of this metal ion, Fe^3+^, is extremely insoluble under physiological pH and can produce toxic oxygen-based radicals via redox cycling in the presence of molecular O_2_.^1^ Living organisms respond to these biochemical limitations by regulating the amount and speciation of iron present within living systems utilizing dedicated high-affinity iron transporters and dedicated iron binding proteins.^1,2^ Due to the importance of these transporters in sustaining life, these have been extensively studied and most known iron transporters from bacterial systems characterized until date rely on ATP/GTP hydrolysis-derived energy or proton motive force (Figure 1).^1,2^

In contrast to these ATP/GTP-dependent iron transporters, iron utilization through FtrABCD is predicted to utilize a ferrous oxidase protein.^3−6^ This is based on the presence of the inner membrane permease, FtrC, that belongs to the ferrous oxidase-dependent Fe^2+^ transporter family.^3,4^ Previous cell studies by our groups on *Brucella abortus* 2308 FtrABCD have confirmed that this system can only utilize unchelated Fe^2+^, the expression of this system is mediated by low iron and acidic conditions, and Fe^2+^ utilization through this transporter is both ATP/GTP and proton motive force-independent.^3^ Similar cell studies from *Bordetella* spp. have shown that all four components are essential for Fe^2+^ utilization by FtrABCD, and the deletion of any of these proteins is detrimental to mutant *Bordetella* under acidic conditions.^4^ Taking these together, it has been proposed that FtrC can utilize Fe^2+^ in a ferrous oxidase-dependent fashion (Figure 1); however, the identity of the ferrous oxidase from this four-component system remains unknown.^3,4^

Although homologues of FtrC permease are abundant in nature, until date only one member from this family has been functionally characterized, the Ftr1p permease from yeast.^7−12^ Data from this yeast system have confirmed that for Ftr1p to be functional, it must coexpress a multicopper oxidase enzyme, Fet3p.^7−12^ These data show that Ftr1p can only translocate Fe^3+^ produced in situ by Fet3p and utilize the free energy released for the transport process as shown below.^7−12^
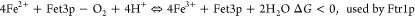


Although Ftr1p and FtrC show high sequence homology and belong to the same permease family,^3−12^ none of the protein components coexpressed with FtrC (FtrA, FtrB, and FtrD) in the bacterial Ftr system are associated with any known redox protein family. A recent in vitro work on a unique five-component FtrAPBCD system from *Rubrivivax gelatinosus* showed Cu^2+^-bound FtrA can oxidize Fe^2+^, making it a possible ferrous oxidase.

On the other hand, we and another group predicted that periplasmic FtrB (10 kDa) is a novel cupredoxin (Cup-II) based on its common ancestry with Fet3p (a multicopper oxidase) and other single-domain cupredoxins.^14,15^ Both multi- and single-domain cupredoxins consist of a protein with a cupredoxin-like fold, which is characterized by two conserved β-sheets formed by parallel–antiparallel β-strands with highly conserved H-bonded structures as well as a well-conserved HHC Cu^2+^ binding site (the Type-1 copper site).^16^ Both the 3D protein structure and the primary coordination shell of these Type-1 copper proteins are essential for their redox activity.^16^ Although FtrB from *Brucella*, *Bordetella*, and *Burkholderia* show a close evolutionary relationship with characterized Type-1 copper site-containing cupredoxins (Cup-III protein),^14,15^ the former does not conserve the HHC residues. Instead, residues D118, H121, E84, E86, and E93 are found to be conserved in all *Brucella* spp. FtrB proteins.^3−5,14^ Structural homology for *Brucella abortus* 2308 FtrB predicts D118 and H121 as potential Cu^2+^ binding residues, whereas E84, E86, and E93 are predicted as potential Fe^2+^/Fe^3+^ binding residues. Taking these together for the FtrABCD system from *Brucella abortus* 2308,^3,13,14^ we propose the following Fe^2+^ oxidation pathway by wild-type Cu^2+^-bound FtrB.
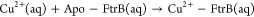

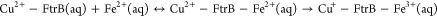


To test the above hypothesis and in continuation to our previous works on this system,^4,13,14^ we conducted experiments on recombinant wild-type FtrB and its two Cu^2+^ binding mutants, D118A and H121A, to investigate their Cu^2+/1+^ (redox cofactor) and Fe^2+^ (redox substrate) affinities, as well as their ability to oxidize Fe^3+/2+^ in vitro. To avoid complication from Fe^2+^, Fe^3+^, and Cu^+^ precipitation and oxidation heat of these metal ions during the experiments, we used their mimics, Mn^2+^, Ga^3+^, and Ag^+^, respectively, in our calorimetric studies.^13^ However, while conducting a spectrophotometric assay to ascertain the ability of Cu^2+^–FtrB to act as a ferrous oxidase, we utilized Fe^2+^. Data from our experiments confirm that wild-type FtrB from *Brucella abortus* 2308 forms an overall cupredoxin-like fold with the typical Greek key β-barrel structure. Our findings also demonstrate that recombinant wild-type FtrB can bind Cu^2+^ and a Fe^2+^ mimic and oxidize Fe^2+^ 3.5-times faster compared to its autoxidation at pH 6.5. To our knowledge, this is the first comprehensive structural and functional characterization of FtrB or any protein within this family under in vitro conditions.

## Results and Discussion

### Diversity in FtrB Evolution

**Figure 2:**
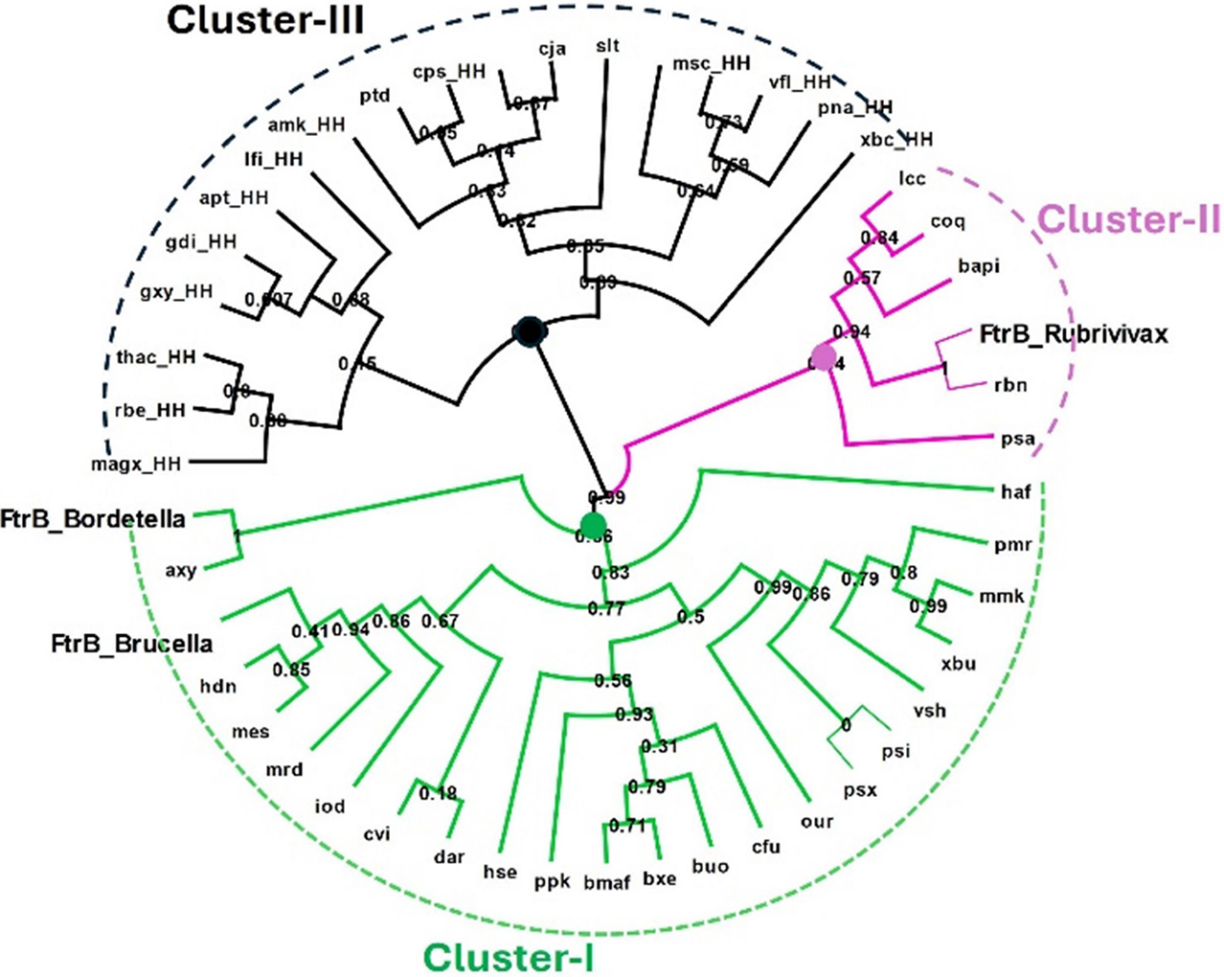
Result of phylogenetic analysis of 14 representative FtrB sequences belonging to either four-component (FtrABCD found in *Brucella, Bordetella, and Burkholderia*) or five-component (FtrAPBCD found in *Rubrivivax gelatinosus*) systems. The tree shows that FtrB from *Rubrivivax gelatinosus* is found in a different cluster (Cluster-II) compared to FtrB from *Brucella* and *Bordetella* FtrB (Cluster III).

In a previous work, we reported a phylogenetic analysis of FtrB from bacteria that encode either the *ftrABCD* or the *ftrABC* genes in an open reading frame,^14^ identifying that the presence of the *ftrD* gene in the operon led to divergent evolution of FtrB. In continuation to that work and based on a recent study reporting a five-component FtrAPBCD system from *R. gelatinosus*, here we present an expanded evolutionary by analyzing FtrB sequences from 14 unique bacteria containing either *ftrABCD* or *ftrAPBCD* in a single open reading frame. Figure 2 shows the results of this analysis, showing that FtrB sequences from the five-component systems appeared in different clades: Cluster II (including *R. gelatinosus* FtrB) and Cluster III, compared to FtrB from four-component systems (Cluster I), such as the one found in *Brucella*, *Bordetella*, and *Burkholderia*. These three clusters are not always phylum-specific, suggesting the presence of some distinct “unknown functional pressure” among bacterial FtrB proteins. We hypothesize that since FtrB from these four organisms (*Brucella*, *Bordetella*, *Burkholderia*, and *R. gelatinosus*) shows divergent evolution, the functions of this protein in these organisms might also differ.

**Figure 3:**
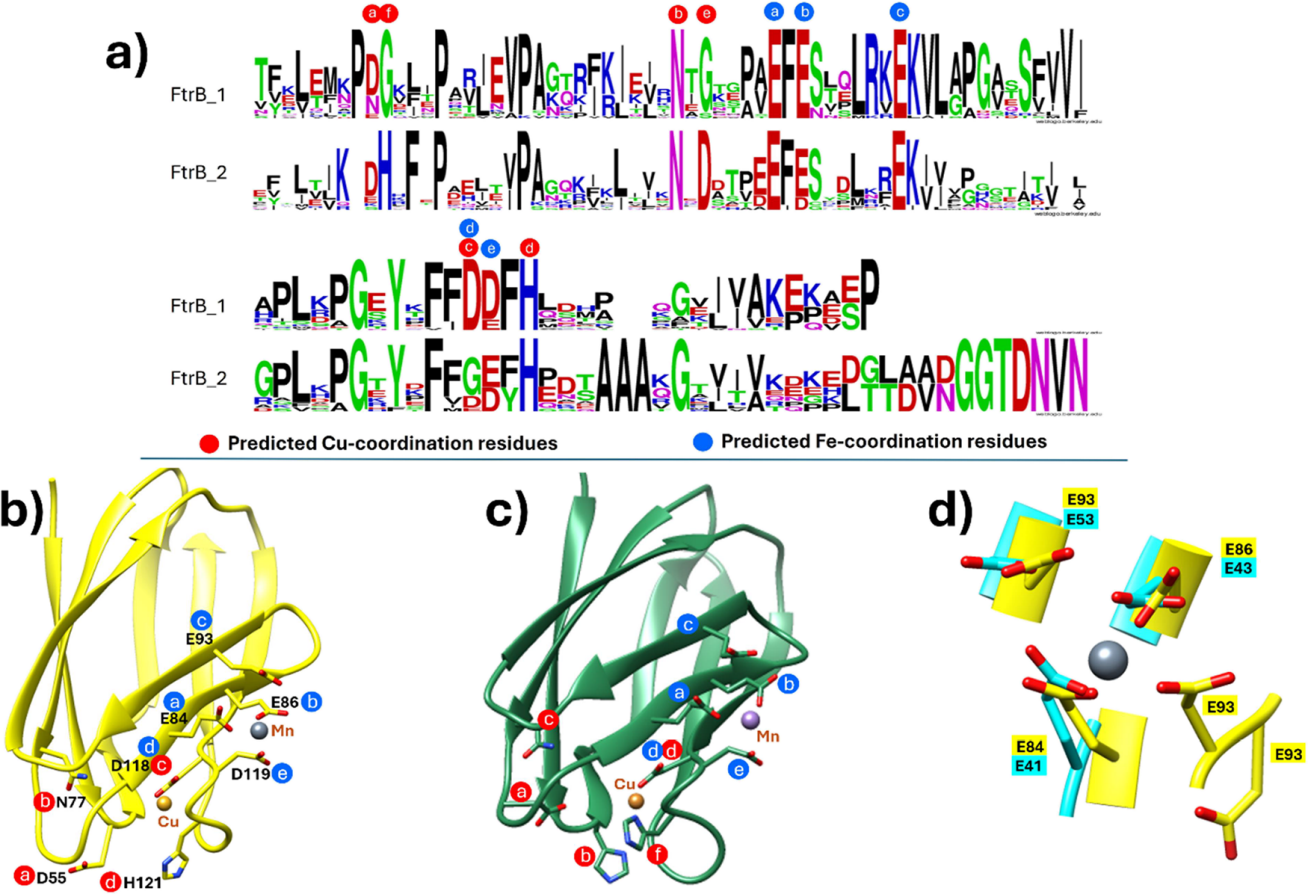
(a) This figure maps the conservation of different residues from the FtrB sequence found in different bacterial systems. FtrB primary sequence is classified into two groups, FtrB Cluster I and FtrB Cluster II, showing different amino acid conservation patterns. Letters in red indicate the possible Cu^2+^ binding residues on FtrB from these two clusters, whereas the letters in blue circles indicate possible Fe^2+^ binding residues. (b, c) Show the crystal structure of *Brucella abortus* 2308 FtrB (8VUK) and the homology model for a Cluster II FtrB and show the putative Cu^2+^ binding sites. (d) This figure shows the Fe^2+^ binding site (we showed a mimic of Fe^2+^, Mn^2+^ in this location) in both FtrB_CI and FtrB_CII family of proteins, and as can be seen, only *Brucella abortus* 2308 FtrB conserves all Cup-II Fe^2+^ binding residues which are also shown to coordinate with Mn^2+^ in its crystal structure.

To gain a better understanding of the amino acid sequence diversity of FtrB from these three clusters, we performed multiple sequence alignment with representative FtrB proteins, and the data from that are presented in Figure 3A. FtrB from all three clusters completely conserves the putative Fe^2+/3+^ binding residues, E84 and E93. The third putative Fe^2+/3+^ binding residue, E86, is partially conserved between the three clusters, being replaced by a D in Cluster III (five-component system). Complete conservation of the putative iron-binding residues indicates this protein’s role in iron sequestration in the periplasm, as predicted.^3−6^

Interestingly, the putative Cu^2+^ binding residues, D118 and H121, from Cluster I (*Brucella abortus* 2308) are not completely conserved between the three clusters (Figure 3A). For example, in Cluster III, D118 is often found as G, losing the crucial carboxylate side chain, which is predicted to coordinate Cu^2+^. Additionally, FtrB from Clusters II and III contains an additional His residue at positions 56 (Cluster III) and 115 (Cluster II), which is not seen in Cluster I FtrB (*Brucella*, *Bordetella*, and *Burkholderia* FtrB).

The Cluster II FtrB homology model (Figure 3c) was generated using AlphaFold (https://alphafold.ebi.ac.uk/), which employs deep learning-based computational methods rather than empirical experimental techniques.

AlphaFold utilizes a combination of theoretical modeling principles, sequence information, and evolutionary constraints to predict protein structures effectively. Superposition of this structure on the crystal structure of wild-type FtrB from *Brucella abortus* 2308 (PDB 8VUK, Figure 3b) shows the additional His residue in the predicted Cu^2+^ binding pocket. Interestingly, DSC data reported recently on FtrB from *R. gelatinosus* (a Cluster II FtrB) showed that this protein did not bind Cu^2+^,^6^ contradicting this prediction about the role of the additional His residue.

### Solution Secondary Structure of FtrB from *Brucella abortus* 2308 Is Dependent on pH, Putative Cu^2+^ Binding Residues, and the Presence of Cu^2+^

**Figure 4:**
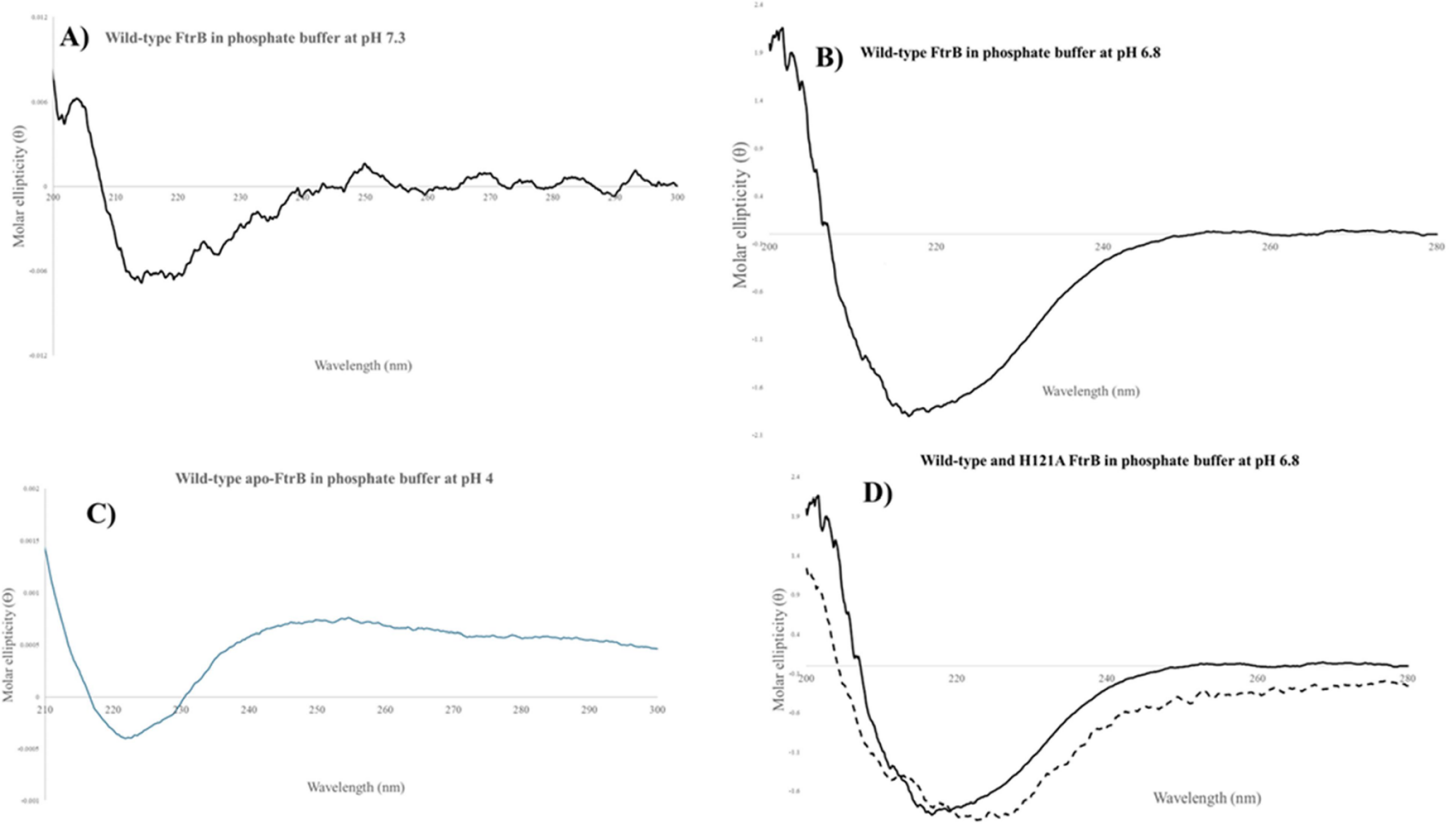
Representative CD spectra for recombinant wild-type and mutant FtrB from *Brucella abortus* 2308 in (A) pH 7.3 phosphate buffer, (B) pH 6.8 phosphate buffer, (C) pH 4.0 phosphate buffer, and (D) CD spectra for wild-type and H121A mutant FtrB in pH 6.8 phosphate buffer.

As mentioned in the Introduction, FtrB is coancestral with known cupredoxins (like rusticyanin) and multicopper ferroxidases.^14,15^ This common ancestry of FtrB, a Cup-II protein, could mean a similar structural fold for this compared to the classical cupredoxins, having a β-sheet-rich Greek key structure. Studies on classical cupredoxins have shown that this structure is essential for their redox enzyme activity.^16^ Homology models of FtrB from *B. abortus* 2308 and *R. gelatinosus* have been reported by us and others, predicting a classical cupredoxin like three-dimensional fold.^6,14^ In this study, we experimentally determined the solution structure of recombinant wild-type and two mutant FtrB proteins (D118A and H121A) under different pH and metalation conditions using circular dichroism (CD) spectroscopy to investigate the validity of such a homology prediction. Normalized CD spectra are presented in Figure 4, with insets showing the percentage of secondary structure composition determined using the BeStSel (Beta Structure Selection) program.^17−19^ The secondary structural elements calculated by this program (Table 1) are categorized into α-helices, parallel β-sheets, antiparallel β-sheets, loops, and other structural motifs.^17−19^

**Table 1:** Estimated Secondary Structural Elements in Wild-Type and H121A Mutant FtrB in Different pH and Buffer Conditions Obtained Using BeStSel^17−19^

protein	2° structure				
	% α-helix	% antiparallel β-sheet	% parallel β-sheet	% turns	% others
wild-type apo-FtrB in DI water	0	34.8	0	17.7	47.5
wild-type apo-FtrB at pH 7.3	0	23.6	31.9	9.5	34.9
wild-type apo-FtrB at pH 6.8	9.8	27.4	20.9	13.2	8.5
wild-type apo-FtrB at pH 4	0.9	44.8	0	14.3	40
wild-type apo-FtrB at pH 7.3 + Cu^2+^	0	52.0	0	16.0	32.0
H121A FtrB at pH 6.8	28.9	35.6	0	35.8	0

The appearance of a single negative peak centered at 217 nm for wild-type FtrB (no added Cu^2+^) in phosphate buffer at pH 7.3 and 6.8 is indicative of the fact that under these conditions, the protein predominantly forms β-sheets (4A and 4B). Analysis of the raw ellipticity data under these conditions using BeStSel showed an increased α-helical fold (9.8%) at pH 6.8 compared with pH 7.3 data (Table 1, Figure 4A,B inset). In contrast to the single peaks obtained for wild-type apo-FtrB (no Cu^2+^ present) at pH 7.3 and 6.8, an additional positive CD peak is observed (at 246) when the protein was present in a pH 4 buffer, which indicates structural alteration under this highly acidic condition. BeStSel analysis of this raw CD data predicts an increased antiparallel β-sheet (44.8%) at this pH compared to those at pH 6.8 and 7.3 (Table 1). Figure 4 also shows CD spectra for apo-FtrB in the presence of Cu^2+^ at pH 7.3, and the appearance of this with Cu^2+^ FtrB spectra is very similar to that of the apo-FtrB (no Cu^2+^) signal. However, BeStSel calculation predicts an increased antiparallel β-sheet structure (52.0%) for wild-type FtrB in contact with Cu^2+^ (Table 1). This alteration of % secondary structure contribution without altering the overall β-sheet fold can be taken as an indication of protein–Cu^2+^ interaction.

In contrast to the wild-type FtrB CD data, the H121A mutant showed a single negative band at 225 nm (Figure 4D), indicative of a global secondary structural change, which was also predicted by a much larger α-helix contribution to this mutant by BeStSel (Table 1). The CD spectrum for the D118A mutant showed increased noise, with the negative peak appearing at 210 nm. Due to the increased noise in this data, we did not perform a BeStSel analysis on this protein. These CD data on FtrB mutants clearly show secondary structural alterations due to mutation of conserved residues predicted to bind with the redox cofactor. Structural changes in proteins can lead to disruption or alteration of their ligand-binding ability.

### Crystal Structure of Recombinant Wild-Type FtrB from *Brucella abortus* 2308 Confirms Cupredoxin-like Fold Formation

**Figure 5:**
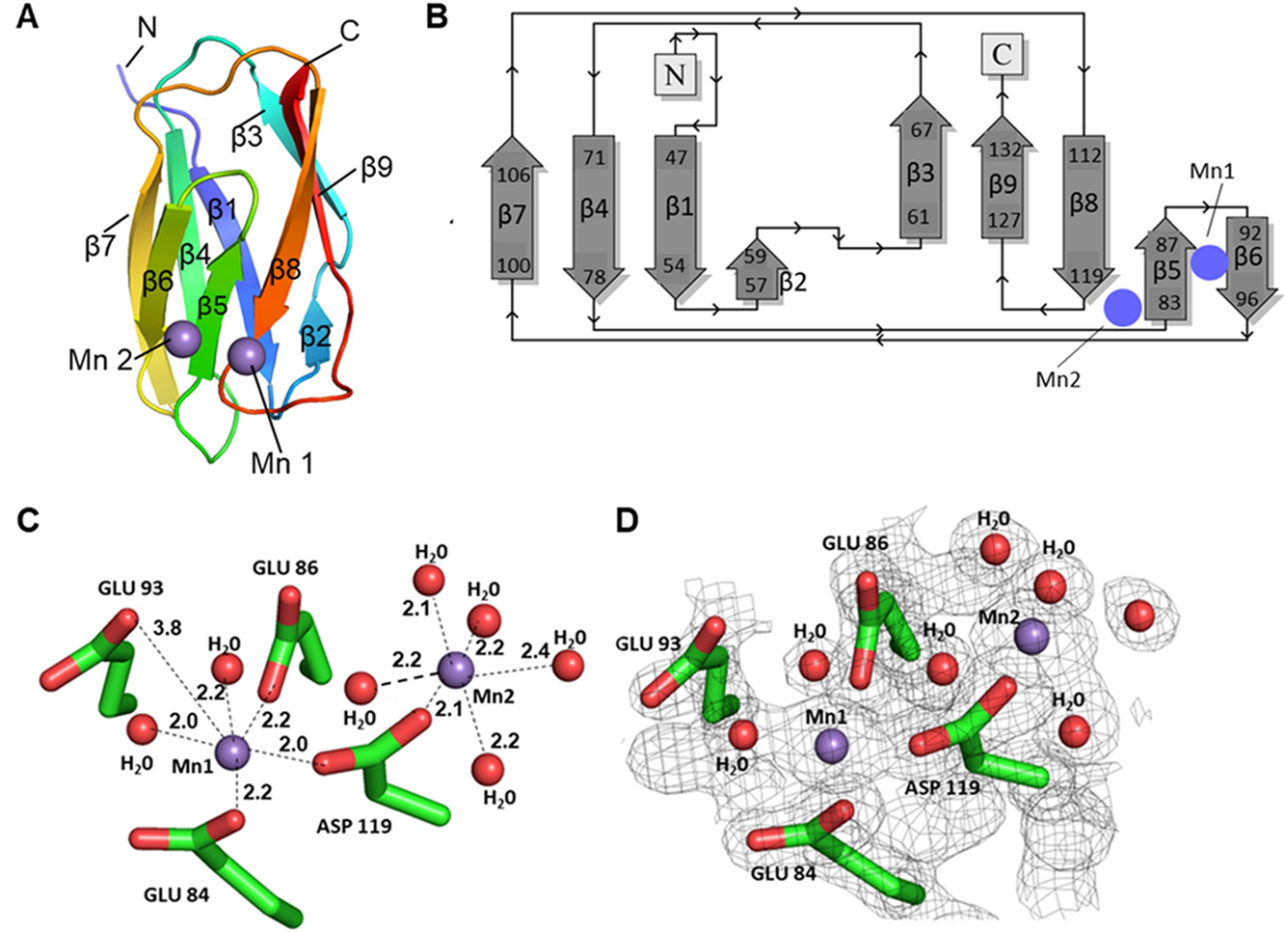
The crystal structure of *B. abortus* FtrB at 1.3 Å. (A) Cartoon representation of *B. abortus* FtrB (colored from the N to C terminus as a spectrum) with Mn shown as violet spheres and coordinating residues shown as sticks. Each of the nine β strands is numbered in order from the N to C terminus. (B) Secondary structure representation of *B. abortus* FtrB produced by PDBsum. β strands are numbered and locations of Mn are depicted as blue circles. (C) Mn (in violet spheres) shown with coordinating residues (Mn1: E84, E86, E93, and D119 and two ordered water molecules) and (Mn2: D119 and five ordered water molecules). Coordinating bonds are drawn with dashed lines, and bond distances are labeled in angstroms. (D) Electron density maps (2Fo–Fc) contoured at 2σ contoured around Mn1 and Mn2. No Cu electron density was detected close to the putative Cu^2+^ binding site (D118 and H121). The p*K*_a_ of H121 in the folded protein is 6.5 and the crystal was grown at pH 4.5 and this might be a reason for not observing Cu^2+^ binding. Amino acid numberings include the 43 amino acid chain signal-peptide as determined by SignalP5.

*Brucella*, *Bordetella*, and *Burkholderia* FtrB were designated as novel cupredoxin (Cup-II), capable of forming a cupredoxin-like fold.^14−16^ However, no experimental proof is available to confirm this evolutionary prediction. We investigated the validity of this phylogenetic prediction by crystallizing recombinant wild-type apo-FtrB from *Brucella abortus* 2308 (see Materials and Methods section for details about making apo-proteins from as-isolated proteins) from 100 mM sodium acetate (pH 4.6), 20% (v/v) polyethylene glycol 3350, and 20–100 mM MnCl_2_ supplemented with 20–100 mM CuSO_4_ and obtained diffraction data at 1.30 Å resolution (Table 2). Following molecular replacement using an AlphaFold2 model, a single copy of FtrB was placed in the asymmetric unit (Figure 5A). The final refined structure (*R*_free_ = 19.8%, Table 2) at a limiting resolution of 1.30 Å was produced.

**Table 2:** X-ray Data Collection and Refinement Statistics for Wild-Type FtrB

parameters	FtrB
PDB ID	8VUK
space group	P1
a, b, c, (Å)	25.811, 27.033, 30.821
α, β, γ, (°)	83.41, 69.64, 71.81
resolution (Å)	28.89–1.30 (1.35–1.30)
*R*_pim_	0.06 (0.159)
*R*_meas_	0.115 (0.277)
C*C*_1/2_	0.962 (0.937)
I/σI	10.9 (4.74)
completeness (%)	94.9 (90.9)
redundancy	3.7 (2.5)
resolution, Å	28.89–1.30 (1.34–1.30)
no. reflections	17,291 (1,198)
*R*_work_/*R*_free_ (%)	17.9/19.8
atoms modeled	842
protein	711
water	129
avg. B-factors (Å)	18.66
protein	16.89
water	28.47
RMSD bond lengths (Å)	0.012
RMSD bond angles (°)	1.44

As can be noted from Figure 5, the crystal structure of *Brucella abortus* 2308 recombinant wild-type FtrB shows an overall cupredoxin-like fold with nine parallel antiparallel β-strands (Figure 5A) as was predicted by evolution data classifying it as a novel cupredoxin (Cup-II) as well as its homology models.^6,14−16^ Topology of the β-strands is shown in Figure 3B which shows a short three-residue β-hairpin between strands 2 and 3. Interestingly, the crystal structure of wild-type FtrB does not show any α-helical component, supporting the secondary structure prediction made by BeStSel at pH 7.3 and 4 using experimental CD data (Table 1). A marked difference between the predicted Cu^2+^ binding site in FtrB compared to the same in characterized cupredoxins with Type-1 residues (HHC) is that in the former, D118 and H121 are in the C-terminal as opposed to the Type-1 residues being found at the N-terminal of characterized cupredoxins.^14−16^

Interestingly, the electron density that can correspond to the presence of copper metal close to the D118 and H121 residues was not identified. This can be due to several reasons, such as the pH of crystallization being 4.6, where the side chain of H121 would be protonated and hinder Cu^2+^ binding (p*K*_a_ for H121 in FtrB using ProPKA 6.5).^21^ Second, acetate being a competitive chelator for Cu^2+^ and being present in such a high concentration under the crystallization condition (100 mM acetate and 10–100 mM CuSO_4_). Finally, the overall charge on wild-type FtrB at this pH is ∼6, compared to an overall negative charge (∼-6) at pH 7.3 (SI Figure 1), and this can hinder the approach of the positive charge. Isothermal titration calorimetry (ITC) data described below show that wild-type FtrB can bind Cu^2+^ under different pH and buffer conditions. Similar observations (absence of Cu^2+^ in the crystal structure but the ability of a protein to bind Cu^2+^ using ITC) have been reported by others as well and have similarly been attributed to crystallization conditions.^22,23^

In contrast, diffraction data from wild-type FtrB crystals showed that two large peaks of unmodeled density above 5σ were found in the Fo–Fc difference map and could be modeled as two Mn^2+^ ions with high occupancy (Mn1 = 0.88 and Mn2 = 0.71) and low B-factors (Mn1 = 15.54 Å2 and Mn2 = 14.20 Å2) (Figure 3C,D). Coordination of Mn1 is mediated by side chain oxygen atoms of residues E84 and E86 (from the EXE motif), E93 (from the RKEKV motif), and D119 and two ordered waters, resulting in octahedral geometry (Figure 5C,D). A second Mn^2+^ (Mn2) is found 5.3 Å from Mn1, near the loop connecting β-strands 5 and 8.^14−16^ Surprisingly, only a single coordinating ligand originates from FtrB (i.e., D119), with five oxygen atoms from ordered water molecules acting as the remaining coordinating ligands, resulting in octahedral geometry (Figure 5C,D). In relation to the overall fold of FtrB, Mn1 is located on the outer surface of FtrB near β-strands 5 and 6, which are separated by a small turn (Figure 5C,D).

**Figure 6:**
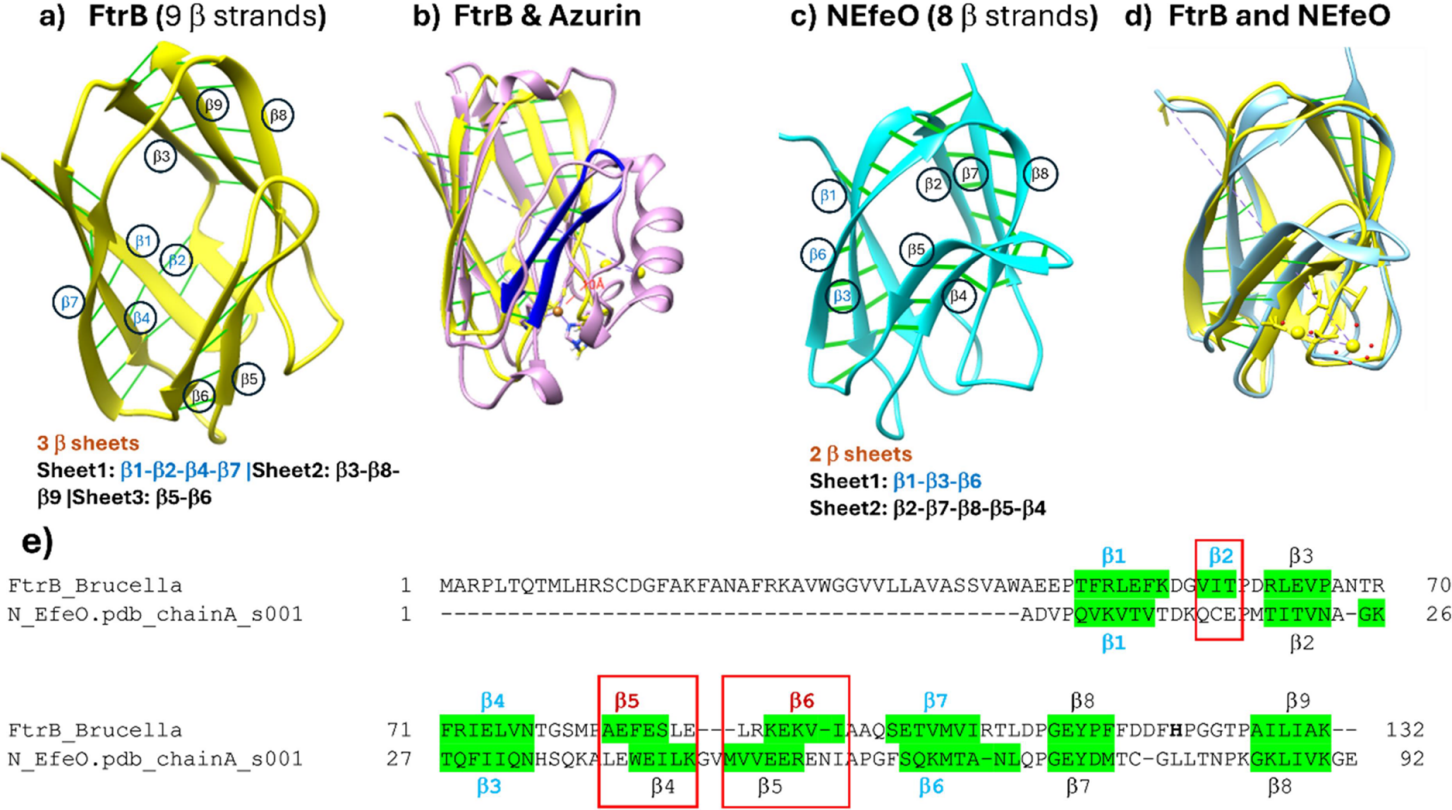
Secondary structural fold comparison using the crystal structures of (a) FtrB; (b) superimposition of the crystal structure of FtrB on azurin; (c) crystal structure of the N-terminal of EfeO; (d) superimposed crystal structures of FtrB and the N-terminal of EfeO. The β strands of the respective structures of FtrB and NEfeO are numbered using Arabic numerals, and H-bonding between strands is depicted by green lines. (e) Structure-based sequence superimposition of FtrB and the N-terminal of EfeO β strands showing differences in lengths.

As the 3D shape and the H-bonding patterns in classical cupredoxins are completely conserved and contribute to their fast electron transfer property,^16^ we conducted a detailed structural analysis using the FtrB crystal structure. We included the crystal structure of FtrB (a Cup-II), classical single domain cupredoxins (Cup-III), and the N-terminal domain of EfeO (Cup-I) to obtain a complete structural understanding for these three coancestral proteins. The result of this analysis revealed striking differences in the number of β-sheets formed by these three families of Cup proteins as well as the H-bond interactions between the β-strands that hold the β-sheets in place (Figure 6a).^24^ For example, structural fold analysis of FtrB revealed the presence of nine β-strands, resulting in three β-sheets through H-bond interactions (Sheet1:1–2–4–7, Sheet2:3–8–9, and Sheet3:5–6). This is in contrast with Cup-III proteins which always form two β-sheets.^16^ Structural superimposition of FtrB with Azurin (*Q* score = 0.41, RMSD = 2.03, % SSE = 67, SI Table 1) reveals that the unique β-Sheet 3 of FtrB is in the vicinity of the Cu-coordinating site of Cup-III proteins. In other words, this analysis shows that FtrB is composed of more β-sheet content than classical cupredoxins (Cup-III). The exact consequence of this greater β-sheet content has yet to be identified.

Crystal structure of *Brucella abortus* 2308 FtrB shows more similarity (*Q* score = 0.67, RMSD = 1.49, % SSE = 100) with the crystal structure of the N-terminal of EfeO from *E. coli* (Figure 6d). However, a closer inspection of these two structures shows some significant differences. For example, FtrB is made of 9 β-strands, whereas the N-terminal of EfeO consists of 8 β-strands. Although both proteins form three β-sheets through H-bonding between these strands (in contrast to two conserved β-sheets in all characterized cupredoxins), the composition and H-bonding patterns within the sheets differ. For example, *Brucella abortus* 2308 FtrB sheet 1 is made of four β-strands (Figure 6a, strands 1, 2, 4, and 7), whereas sheet 1 from the N-terminal of EfeO is made of three β-strands (Figure 6c, 1, 6, 3). Further, the β-strands in sheet 1 from FtrB are arranged in an antiparallel and parallel fashion, whereas the same in the N-terminal of EfeO are all antiparallel to each other. The rest of the two sheets from both proteins consist of antiparallel strands. Finally, the length of β-strand 5 from sheet 3 in the N terminal of EfeO is longer than that of β-strand 5 from sheet 3 in FtrB.

These finer structural differences between Cup-I, II, and III proteins are significant, given prior experimental data on several Cup-III proteins have shown the rates of electron transfer being correlated to the H-bonding network as well as the composition of the β-sheets in these proteins.

### Heat Capacity vs Gibb’s Free Energy Graphs for Recombinant Wild-Type FtrB Show Buffer and Cu^2+^-Dependent Variations

**Figure 7:**
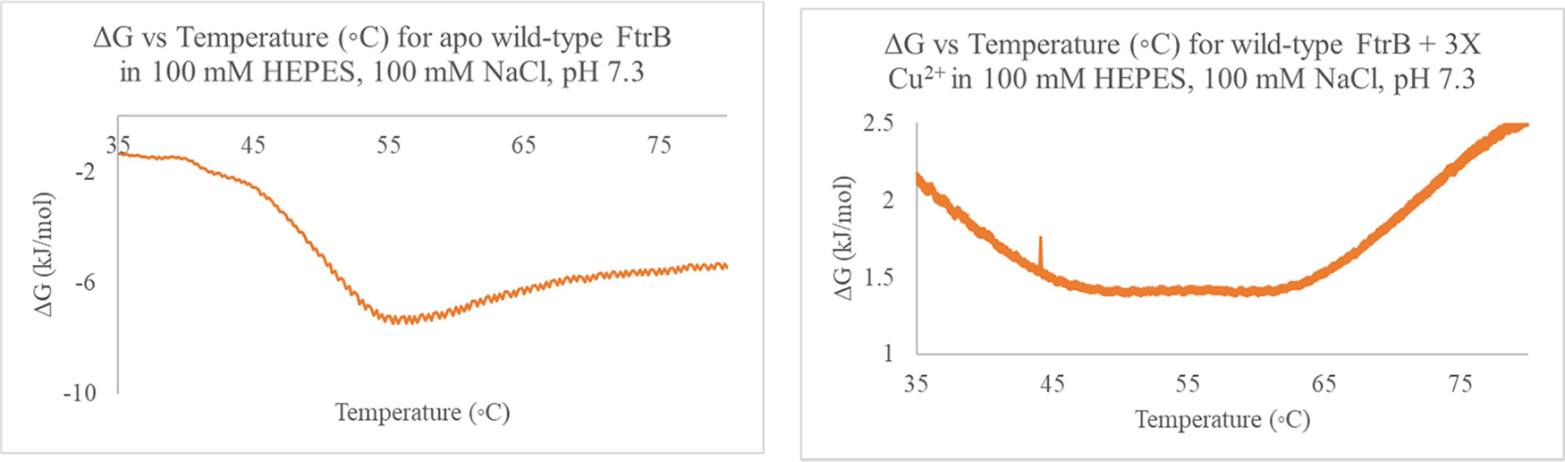
Representative Δ*G* vs temperature plots for recombinant wild-type FtrB under different conditions. Δ*G* was calculated using buffer subtracted raw heat capacity data without using any model, Δ*H* = Σ(*T*_*i*+1_–*T_i_*), Δ*S* = Σ(*T*_*i*+1_–*T_i_*), and Δ*G* = Δ*H*–*T_i_* Δ*S*.^25^ The Δ*G* versus temp peak is much broader in the presence of Cu^2+^, and this can be due to multiple folded structure in the presence of the metal ion.

We performed DSC experiments on wild-type and mutant FtrB in the absence and presence of metal ions to determine the change in heat capacity (*C*_p_) and Δ*G* over the scan temperature range studied (Figure 7). The raw heat data obtained was processed without using any data fitting model to obtain thermodynamic parameters for protein unfolding using methods described by others earlier.^25^ Heat capacity differences between the folded and unfolded wild-type FtrB (Δ*C*_p_) showed the usual positive sign under all buffer conditions, indicating a more open linearized form for the protein upon unfolding. However, the magnitude of this Δ*C*_p_ difference varied (Δ*C*_p_ ∼ 4.5 kJ/K mol in 100 mM HEPES, 100 mM NaCl, pH 7.3; Δ*C*_p_ ∼ 8.0 kJ/K mol in 100 mM Tris.HCl, 300 mM NaCl, 20 mM imidazole, pH 7.4 or the His-wash buffer; and Δ*C*_p_ ∼ 3.0 kJ/K mol) in different buffer systems, indicating either the structure of the unfolded state or the structures of both the folded and unfolded states show buffer dependence.^23^ This difference in Δ*C*_p_ and consequently protein folding stability is attributed to the varying concentrations Cl^–^ in the buffers as predicted by the Hofmeister effect.^26^

Plots for Δ*G* for unfolding versus the scanning temperature range are presented in Figure 7. As can be noted, the wild-type apoprotein in 100 mM HEPES, 100 mM NaCl, pH 7.3 shows a negative Δ*G* peak around 55 °C, whereas in the presence of Cu^2+^ (3-times excess), the Δ*G* value is positive throughout the scanning temperature range with a very broad and shallow Δ*G* peak between 45 and 65 °C. We interpret this positive Δ*G* vs temperature graph in the presence of Cu^2+^ as an indication of the stabilization of the folded protein as well as the existence of multiple folded structures in that temperature range. A recent report used DSC on FtrB from another organism, showing the addition of Cu^2+^ did not alter the fitting model-dependent Tm for the protein. Our model-independent data analysis is in contrast with that data and clearly show apo-FtrB unfolding is more spontaneous than when Cu^2+^ is present with the wild-type protein (Figure 7).^25^ This difference in data could either arise from the fact that the FtrB proteins from these two organisms evolved differently and/or the unreliability of the fitting model.^25^ Divergent evolution between FtrB from these two organisms has been discussed earlier in this paper (Figure 2).

### Recombinant Wild-Type FtrB Binds Cu^2+^ and a Fe^2+^ Mimic Forming the Active Enzyme–Substrate Complex

For *Brucella abortus* 2308 FtrB to perform the function of Fet3p, a multicopper oxidase, in FtrC-mediated iron transport, it was predicted that this periplasmic protein binds to Cu^2+^ using conserved residues D118 and H121. Further, to act as a ferrous oxidase, Cu^2+^–FtrB must also form an enzyme–substrate complex, Cu^2+^–FtrB–Fe^2+^. We performed ICP-MS and calorimetric investigations to determine these predicted metal affinities and the roles of conserved residues in such functions, thus allowing the transition metal ion to act as the redox cofactor.

ICP-MS experiments on as-isolated (without dialyzing with a chelator) wild-type FtrB were performed in 25 mM ACES at pH 7.3, and experimentally determined % saturation of this protein (after buffer contribution subtraction) is presented in Table 3 and SI Table 2. As can be seen, the as isolated protein purifies as 71% saturated with copper, assuming 1:1 Cu^2+^: FtrB binding. We also tested this protein for the presence of Fe and Ni, and under our experimental conditions, these elements were not detected from the as-isolated protein solution. Recombinant wild-type FtrB is exposed to different nutrients, including micronutrients like Cu, Mn, Fe, Ni, etc., during its expression and purification steps. Given that this protein showed the presence of only Cu in ICP-experiments indicates specific interaction between recombinant wild-type FtrB from *Brucella abortus* 2308. Since copper is a micronutrient present in the growth medium, it is likely that the recombinant wild-type protein acquired this metal ion during growth. We also checked for the presence of iron, manganese, and cobalt in the isolated wild-type protein, but none of these metals were detected.

**Table 3:** Data Table Showing the % Cu^2+^ Saturation in As-Isolated Wild-Type FtrB Using ICP-MS^a^

Cu^2+^ in As-isolated wild-type FtrB	10^–6^ g/mL
expected	1.02
experimental	0.719
Cu % saturation	71

a The g/mL values are calculated by converting the experimentally obtained ^63^Cu ppb values after performing dilution correction and subtraction of the buffer blank (25 mM ACES, pH 7.3). The % of Cu^2+^ in the As-isolated protein  is calculated with the assumption that wild-type FtrB can bind Cu^2+^ in a 1:1 ratio ([As-isolated wild-type FtrB] = 16 μM).

**Figure 8:**
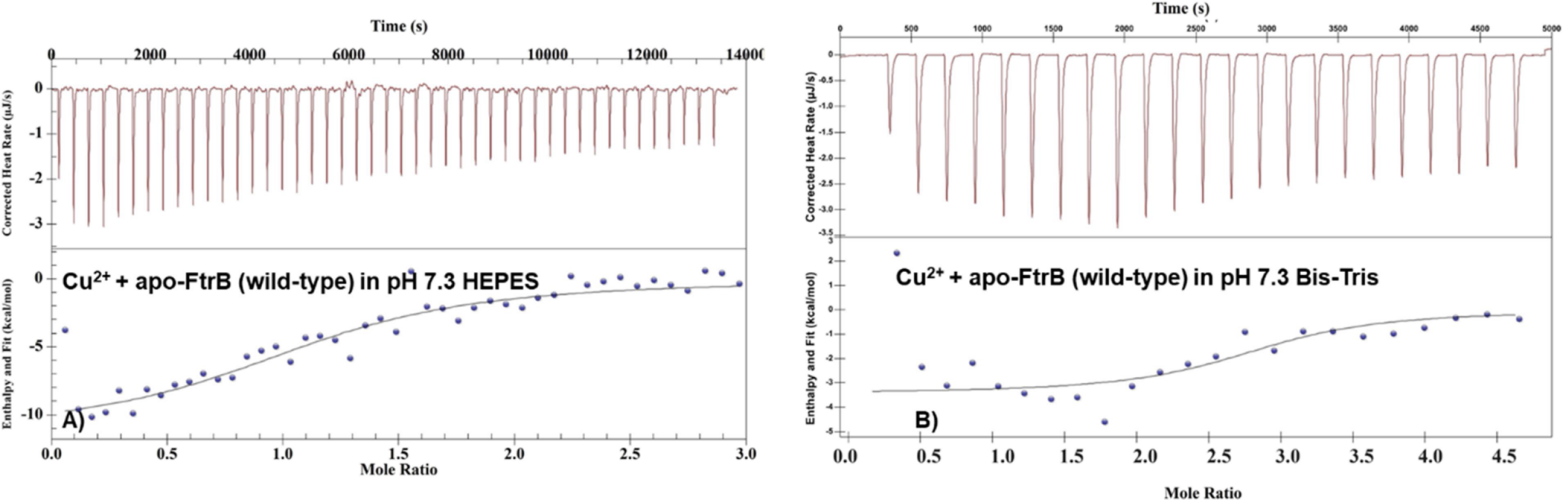
Representative ITC thermograms for (A) Cu^2+^ titrated into wild-type apo-FtrB in 100 mM HEPES, 100 mM NaCl, pH 7.3; (B) Cu^2+^ into H121A apo-FtrB in 100 mM HEPES, 100 mM NaCl buffer, pH 7.3. In all experiments, the concentration of the protein was 50 μM, and the sample and reference cells were maintained at 25**°**C. The upper part of each thermogram shows the heat generated during incremental ligand addition, and the lower half shows the integrated heat data (as dots) with the best fit shown with thin lines. The raw exothermic heat data was integrated, and the best fit of this data was obtained by using an independent single site binding model.

**Figure 9:**
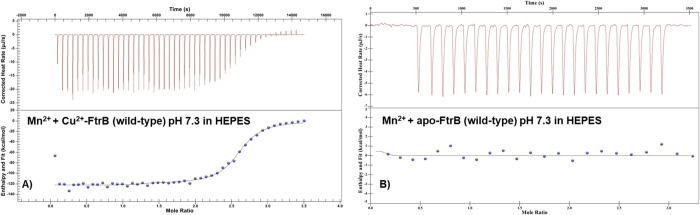
Representative ITC thermograms: (A) Mn^2+^ titrated into wild-type Cu^2+^–FtrB in 100 mM HEPES, 100 mM NaCl, pH 7.3; (B) Mn^2+^ into wild-type apo-FtrB in 100 mM HEPES, 100 mM NaCl buffer, pH 7.3. In all experiments, the concentration of the protein was 50 μM, and the sample and reference cells were maintained at 25 °C. The upper part of each thermogram shows the heat generated during incremental ligand addition and the lower half shows the integrated heat data (as dots) with the best fit shown with thin lines. The raw exothermic heat data was integrated, and the best fit of these data was obtained by using an independent single site binding model.

*Brucella abortus* 2308 FtrB is predicted to bind Cu^+^, Cu^2+^ and oxidize Fe^2+^ to Fe^3+^ according to the equations shown in the Introduction. To avoid heat of precipitation and/or oxidation under our experimental conditions, we conducted ITC experiments using mimics for Fe^2+^ (Mn^2+^), Cu^+^ (Ag^+^), and Fe^3+^ (Ga^3+^) as discussed before.^13^ Experimental data from these titrations are tabulated in Table 4, and representative thermograms are presented in Figures 8 and 9. The wild-type apo-FtrB protein showed μM Cu^2+^ binding affinities in 100 mM MES, 100 mM NaCl, pH 7.3 and 100 mM HEPES, 100 mM NaCl, pH 7.3 at 25 °C (*K*_d_ of 23.3 ± 5.1 and 0.32 ± 0.02 μM, respectively) with exothermic binding heats (Figure 8, Table 4). The binding enthalpy from ITC experiments (Δ*H*_ITC_) and other thermodynamic parameters are a sum of all enthalpic processes taking place during the protein–ligand binding event (Δ*H*_ITC_ = +Δ*H*_Buffer-Cu2+_ – Δ*H*_Protein-Cu2+_ + Δ*H*_apoprotein Cu2+binding site dehydration_ + ...) and depends on the buffer contributions, which can be taken as a justification for differences in FtrB-Cu^2+^ affinities.^27^ ITC data for Cu^2+^ into wild-type apo-FtrB in Bis-Tris buffer at 25 °C showed a high signal-to-noise ratio; however, when this experiment was performed at 50 °C (right below the temperature where Δ*G* unfolding is most negative for the apo protein, Figure 7), the wild-type protein was able to bind Cu^2+^ with 3.0 ± 1.0 μM affinity. These experiments, taken together with the data from CD, ICP-MS, and DSC, clearly show that recombinant wild-type FtrB can bind Cu^2+^ under various experimental conditions.

**Table 4:** Thermodynamic Parameters from ITC Experiments on Recombinant Wild-Type and Mutant FtrB from *Brucella abortus* 2308^a^

protein	buffer (pH, °C)	metal	*K*_d_ (μM)	Δ*H*_ITC_ (kcal/mol)	*n*
wild-type apo-FtrB	100 mM HEPES, 100 mM NaCl (7.3, 25 °C)	Cu^2+^	23.3 ± 5.1	–11.82 ± 1.01	1.24 ± 0.08
wild-type apo-FtrB	50 mM Bis-Tris (pH 7.3, 50 °C)	Cu^2+^	3.0 ± 1.0	–3.64 ± 0.43	2.15 ± 0.50 (2)*
wild-type apo-FtrB	100 mM MES, 100 mM NaCl (7.3, 25 °C)	Cu^2+^	0.32 ± 0.02	–23.8 ± 10.5	0.8 ± 0.05
wild-type apo-FtrB	100 mM HEPES, 100 mM NaCl (4, 25 °C)*	Cu^2+^	0.30 ± 0.4	–0.46 ± 0.16	2
			19.2 ± 5.7*	–7.62 ± 0.57*	
wild-type apo-FtrB	100 mM HEPES, 100 mM NaCl (7.3, 25 °C)	Mn^2+^	not reproducible		
wild-type Cu^2+^–FtrB	100 mM HEPES, 100 mM NaCl (7.3, 25 °C)	Mn^2+^	2.13 ± 0.46	–63.0 ± 0.05	2.33 ± 0.41
wild-type apo-FtrB	DI water (25 °C)	Ag^+^	3.47 ± 0.7	–105 ± 30	1.10 ± 0.30
wild-type Ag^+^–FtrB	DI water (25 °C)	Ga^3+^	12.08 ± 0.01	28.94 ± 10	2.84 ± 0.46
H121A FtrB	100 mM HEPES, 100 mM NaCl (7.3, 25 °C)	Cu^2+^	24.2 ± 11.5	–8.001 ± 4.47	1.75 ± 0.57
D118A	100 mM HEPES, 100 mM NaCl (7.3, 25 °C)	Cu^2+^	no binding		

a Calculated parameters with * were obtained using a second program, affinimeter, by fitting single ITC data. The raw heat data could only be modeled with this program if a two independent site model is used.

Currently, we are studying the kinetics of interaction between apo wild-type FtrB in pH 4 100 mM HEPES, 100 mM NaCl with Cu^2+^ using incremental ITC, and our preliminary data showed two heat events upon titration of Cu^2+^ at 25 °C. More detailed investigation is required to understand the origin of these two events, but we hypothesize that the lower exothermic Δ*H* (−0.46 ± 0.16 kcal/mol) is associated with protein structural reorganization in solution prior to Cu^2+^ binding and/or lower buffering effect of HEPES at this pH. This is in agreement with our CD data which showed altered % secondary structure contribution at this pH (Table 1) as well as the fact that in the solid-state structure (lacking the reorganization), no Cu^2+^ was found in the predicted copper binding site. The *K*_d_ associated with this step (Table 4) is the equilibrium constant between the concentrations of conformation that can bind Cu^2+^ to the one that cannot (*K*_d_ 0.30 ± 0.4 μM). The larger exothermic heat event Δ*H* (−7.62 ± 0.57 kcal/mol) is assigned to Cu^2+^ binding with a *K*_d_ 19.2 ± 5.7 μM, like the value in this buffer at pH 7.3.

The ability of Cu^2+^–FtrB (wild-type) to form the enzyme–substrate complex (Cu^2+^–FtrB–Fe^2+^) was confirmed by titrating Mn^2+^ (a Fe^2+^ mimic) into a solution of Cu^2+^–FtrB at 25 °C in 100 mM HEPES, 100 mM NaCl, pH 7.3. Heats generated by injecting Mn^2+^ into wild-type apo-FtrB in 100 mM HEPES, 100 mM NaCl, pH 7.3 were not reproducible in magnitude or sign (Figure 9B); however, like its periplasmic partner FtrA,^13^ when Mn^2+^ was titrated into wild-type FtrB containing 1:1 protein, Cu^2+^ showed reproducible binding data with *K*_d_ 2.13 ± 0.46 μM and *n* ∼ 2 (Table 4, Figure 9A). The crystal structure of wild-type FtrB also showed two Mn^2+^ bound to it using E84, E86, and E93 residues, and this ITC data agrees with that (Figure 3).

### Recombinant Wild-Type FtrB Requires the Conserved Cup-II Residue, D118, for Cu^2+^ Binding

**Figure 10:**
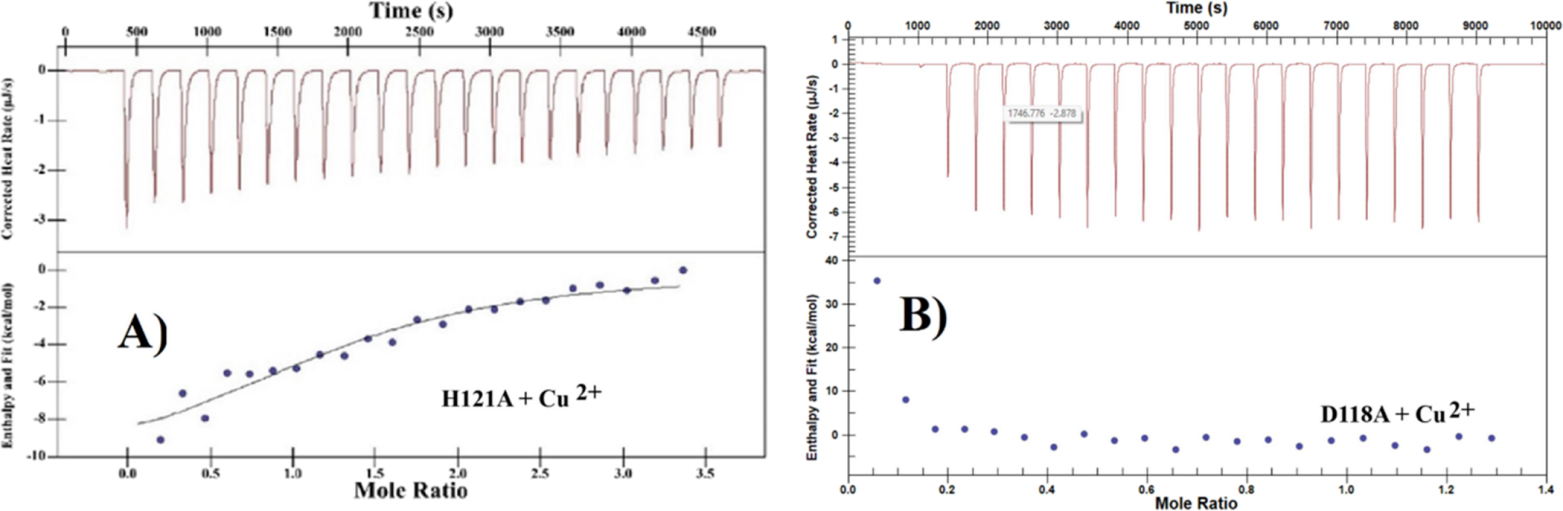
Representative thermograms for Cu^2+^ titration in (A) H121A and (B) D118A FtrB in 100 mM HEPES, 100 mM NaCl, pH 7.3. The upper portions of the thermograms show raw exothermic heat data upon Cu^2+^ titration, and the bottom panels show the integrated heat data (as dots) and the line of best fit (as solid line).

We also performed Cu^2+^ ITC experiments on D118A and H121A mutants of FtrB, as these residues are predicted to bind Cu^2+^.^14,15^ The H121A mutant showed Cu^2+^ affinity like the wild-type protein, but comparison of Δ*H*, Δ*S*, and Δ*G* for Cu^2+^ binding to wild-type FtrB and H121A (Figure 10A and SI Figure 3, Table 4) revealed significant differences. D118A did not show Cu^2+^ binding heat in our ITC experiments (Figure 10 B, Table 4), indicating that this residue plays a crucial role in Cu^2+^ binding. Combined with the CD data for H121A, we conclude that Cu^2+^ might bind at a location different from that in this mutant than in the wild-type protein. We also performed ITC experiments on an FtrB mutant that contained both D118 and H121 residues but lacked a conserved acidic residue (D55) as a control experiment. This D55A mutant showed similar to the wild-type Cu^2+^ affinity and stoichiometry (*K*_d_ = 8.00 ± 3.03 μM, *n* = 1.5), indicating that Cu^2+^ binding to FtrB is a specific event (data not shown).

**Figure 11:**
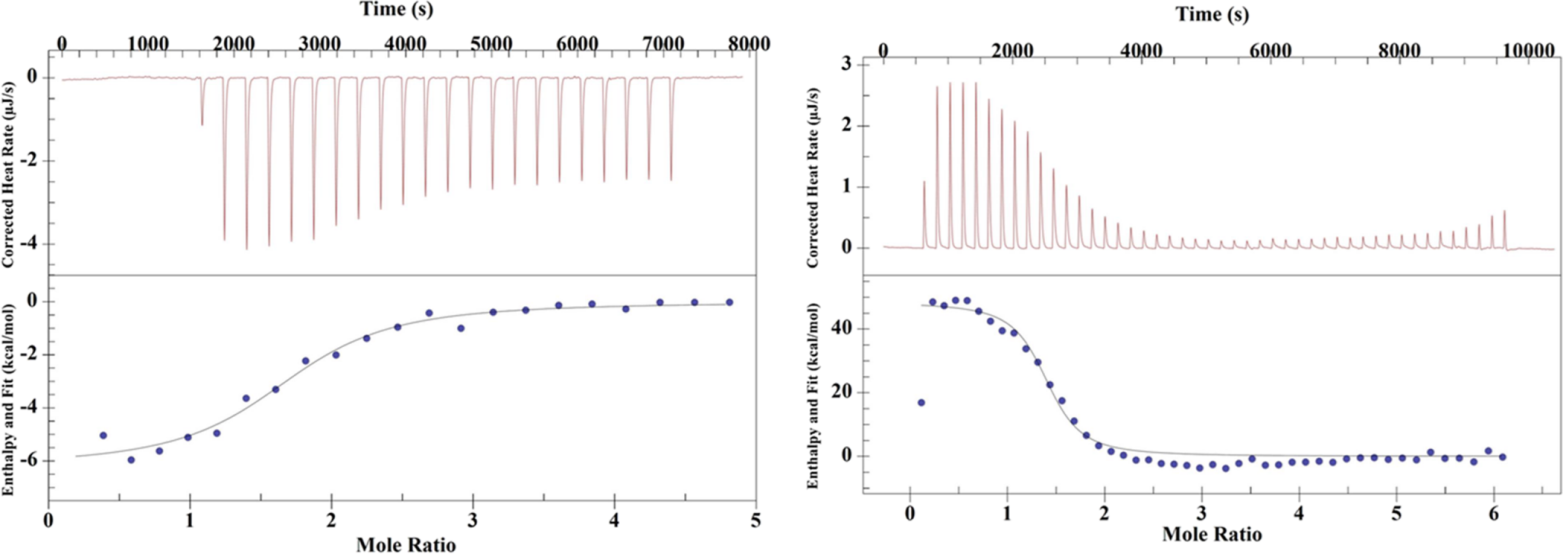
Representative thermograms for (A) Ag^+^ and (B) Ga^3+^ titrations into wild-type apo-FtrB and Ag^+^–FtrB, respectively, in degassed DI water. The upper portions of the thermograms show raw exothermic heat data upon Cu^2+^ titration, and the bottom panels show the integrated heat data (as dots) and the line of best fit (as solid line).

As a ferrous oxidase enzyme using bound Cu^2+^ as a redox cofactor, FtrB is required to stay bound to Cu^+^, in addition to Cu^2+^. Moreover, upon oxidizing its substrate, Fe^2+^, the reduced enzyme (Cu^+^–FtrB) must stay bound to Fe^3+^ as the loss of Fe^3+^ from the reduced enzyme will cause precipitation and redox toxicity (Reactions 1a and 1b). We performed ITC experiments using the nonredox active Ag^+^ and Ga^3+^ as mimics for Cu^+^ and Fe^3+^, respectively, to test these hypotheses. As Ag^+^ can precipitate out in the presence of buffer components, these ITC experiments were performed in neat DI water. Representative ITC thermogram for Ag^+^ binding to wild-type apo-FtrB is presented in Figure 9 showing low 3.47 ± 0.7 μM affinity. Ga^3+^ also showed 12.08 ± 0.01 μM affinity when it was titrated into a solution of Ag^+^–FtrB (Figure 11, Table 4). The affinity of the Fe^3+^ mimic to FtrB is around six times weaker than its affinity for the Fe^2+^ mimic (Table 4). While this should be interpreted with caution, it aligns with the expectation that Fe^3+^ must be released from FtrB to facilitate its translocation through FtrC.^4,5^ These experiments conclusively show that recombinant wild-type FtrB can bind to the required oxidation states of both its redox cofactor and its redox substrate.

### Redox Property and Kinetics of Fe^2+^ Oxidation by Recombinant Wild-Type FtrB

**Figure 12:**
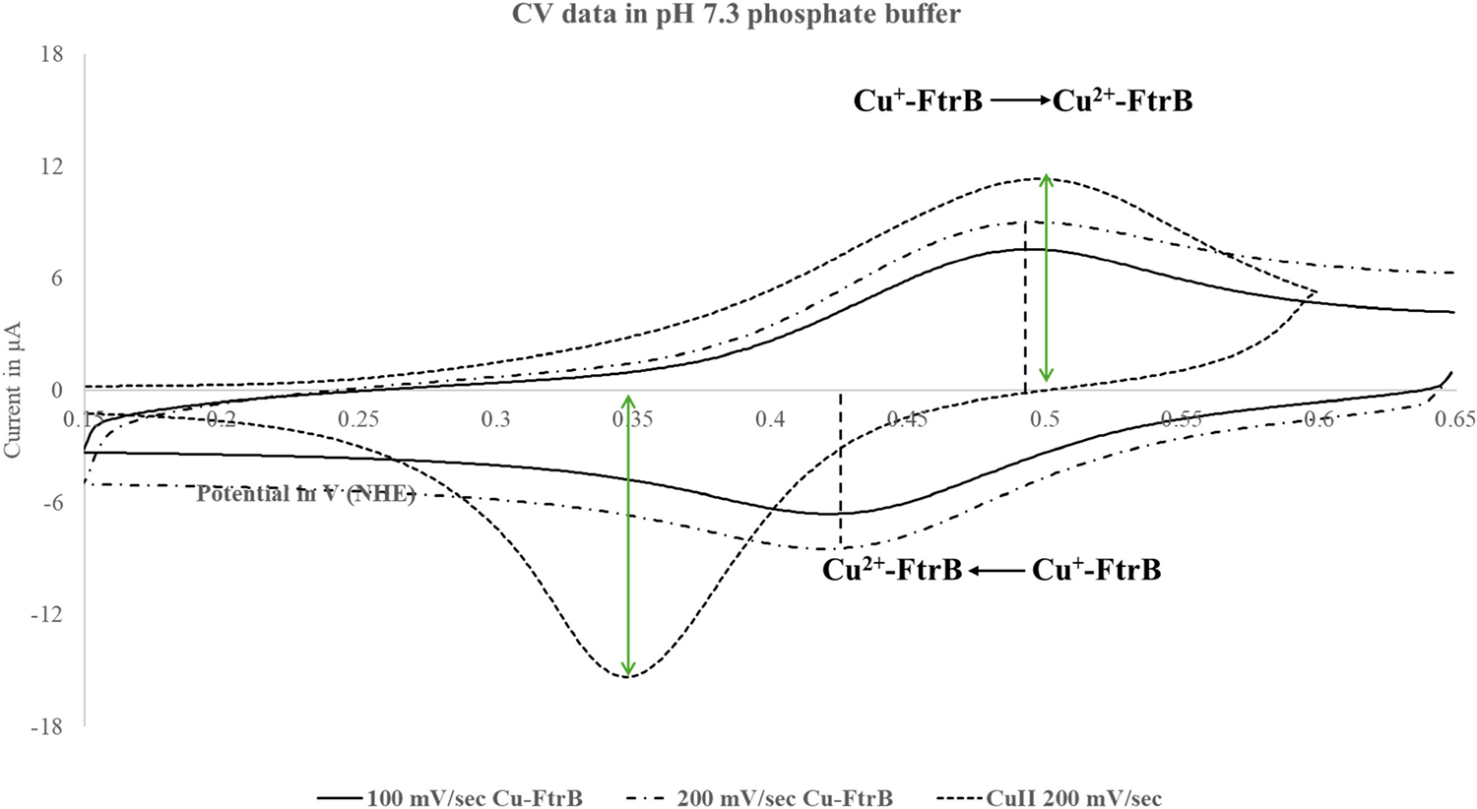
Representative voltammograms for wild-type as-isolated FtrB in phosphate buffer at pH 7.3 ran at two different scan rates (100 and 200 mV/s). The current vs potential sweep data for aqueous solution of copper(II) sulfate in phosphate buffer, pH 7.3, is shown as dashed line, and the peaks are marked by a green double headed arrow. The difference between wild-type FtrB oxidation and reduction potentials (Δ*E* ∼ 50 mV) falls within the reversible redox process, indicating that the molecular orbital energy levels in the oxidized and reduced species are not altered appreciably due to the one-electron redox process.

The ability of Cu^2+^-bound FtrB to oxidize Fe^2+^, as shown in Reaction 1b, requires the Cu^2+^ cofactor to have a reduction potential within the biological window (stability of water zone, + 1 to −1 V vs NHE). To investigate the redox potential of Cu^2+^ bound to wild-type FtrB, we performed cyclic voltammetry (CV) experiments using a three-electrode method, where the gold working electrode was modified by forming a self-assembled monolayer of the protein. We conducted CV experiments on as-isolated wild-type FtrB in two different buffers, given the ICP-MS data indicating ∼70% copper saturation. A representative voltammogram for recombinant as-isolated wild-type FtrB is shown in Figure 12, and the redox data are presented in Table 5. As seen, recombinant wild-type Cu^2+^–FtrB in pH 7.3 phosphate buffer underwent reduction at 477 mV, and the reduced species were reoxidized at 523 mV, making the redox process reversible (Δ*E* = 46.0 mV) with a midpoint potential of 500 mV. The reversibility of redox processes indicates minimal changes in molecular structure upon oxidation and reduction, resulting in electron acceptance and donation within a narrow potential difference. It is also important to note that although the midpoint potential for as-isolated FtrB in both buffer systems was similar, the oxidation and reduction potentials showed buffer dependence, with the redox process being quasi-reversible in 100 mM MES at pH 7.3. Finally, similar CV experiments on apo-H121A FtrB incubated with Cu^2+^ (2×) were performed (Table 5). Both oxidation and reduction potentials for this protein in phosphate buffer were shifted to more negative values, and the redox process was irreversible (Δ*E* = 97 mV). These differences can be attributed to variations in the Cu^2+^ binding environment in H121A compared to the wild-type protein, as suggested by CD and ITC experiments. The *E*_1/2_ for wild-type FtrB is within the biological redox window and is like another ferrous oxidase, rusticyanin, belonging to the Cup-III family of proteins.^14−16^

**Table 5:** CV Data Were Acquired in MES and Phosphate Buffer at pH 7.3 Using a Pt Counter Electrode, a Ag/AgCl Reference Electrode, and a Au Electrode Modified with MPA^a^

protein	buffer (pH)	*E*_R_ (mV)	*E*_O_ (mV)	Δ*E* (mV)	*E*_1/2_ (mV)
As-isolated FtrB	100 mM MES (7.3)	430	501	71	465
As-isolated FtrB	100 mM phosphate (7.3)	477	523	46	500
H121A	100 mM phosphate (7.3)	376	473	97	

a The Ag/AgCl counter electrode was calibrated using ferrocyanide/ferricyanide CV and showed a 39.875 mV drift. The data below are in NHE scale, and the drift has been accounted for. The following data are for 200 mV/s scan rate.

**Figure 13:**
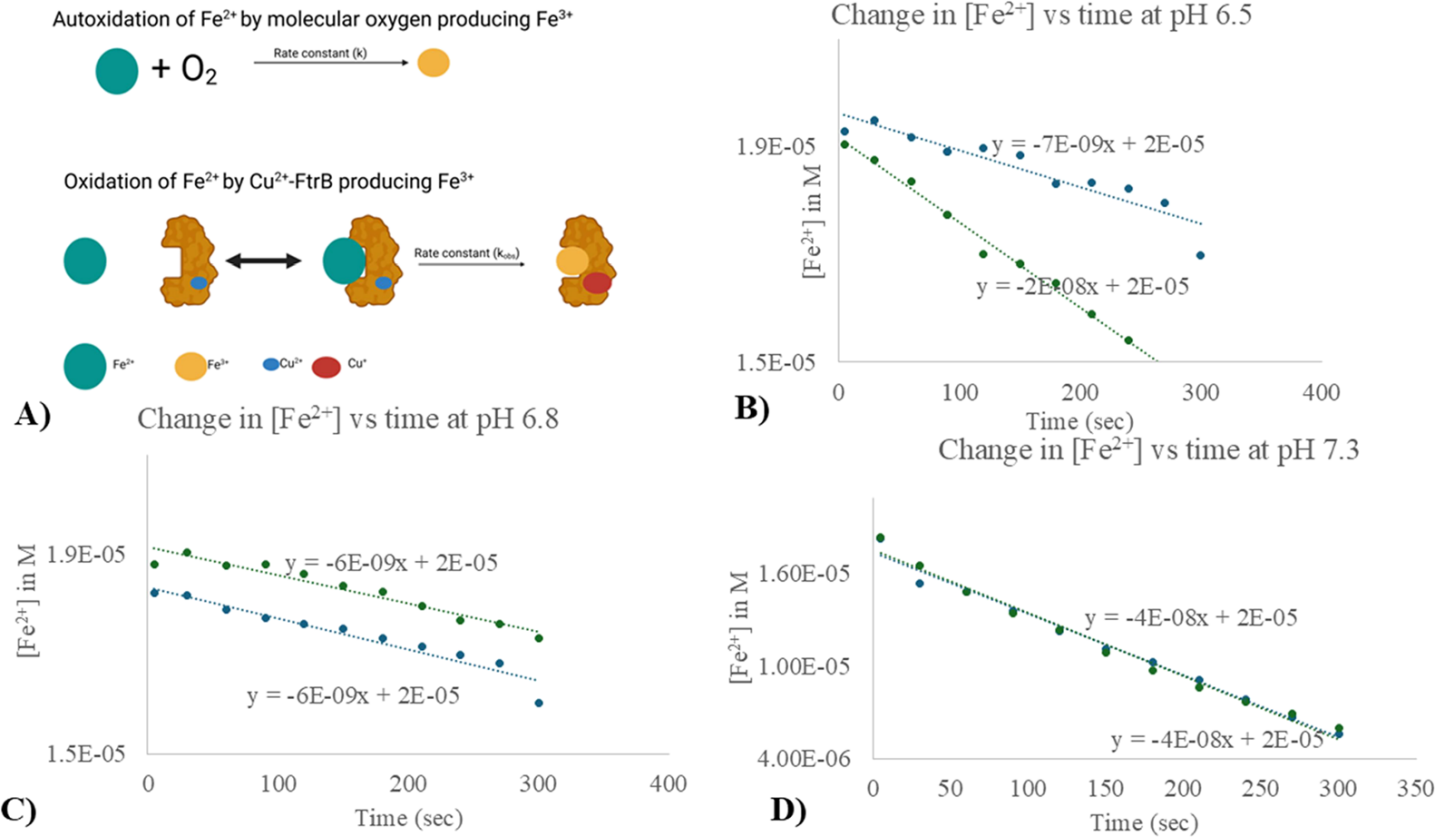
(A) Cartoon representation of the two reactions, autoxidation of Fe^2+^ by dissolved oxygen and the enzymatic oxidation by Cu^2+^–FtrB. This scheme is based on ITC experiments show that the Fe^2+^–FtrB–Cu^2+^ complex has 2.13 ± 0.46 μM *K*_d_ (Table 4). ITC experiments also show that a Fe^3+^ mimic stays bound to wild-type FtrB (*K*_d_ 12.08 ± 0.01 μM). (B–D) Show change in [Fe^2+^] with time at pH 6.5, 6.8, and 7.3, respectively. The green dots show the experimentally determined [Fe^2+^] at that time when Cu^2+^–FtrB was present in the solution, whereas the blue dots show the same when no Cu^2+^–FtrB was present. The dashed lines are the lines of best fits which are also represented by the equations on top of the lines. These data show that the rate of change in [Fe^2+^] is faster when Cu^2+^–FtrB is present only at pH 6.5 (Figure 13B, steeper slope). At the other two pH tested, the rate of Fe^2+^ loss due to autoxidation and in the presence of Cu^2+^–FtrB are identical. The conditional rate for Fe^2+^ oxidation at pH 6.5 by Cu^2+^–FtrB is 3-times faster than the autoxidation of this metal ion.

Since the reduction potential of recombinant wild-type Cu^2+^–FtrB falls within the biological redox window, we performed a spectrophotometric assay to determine the decrease in Fe^2+^ concentration in the presence of wild-type and D118A mutant FtrB at three different pH values under aerobic conditions. As Fe^2+^ can autoxidize in the presence of O_2_, we performed identical control experiments in the tested buffers with no wild-type protein or denatured FtrB (heated at 95 °C prior to adding Fe^2+^). To determine changes in [Fe^2+^] concentrations over time, reaction mixtures (containing known initial concentrations of Fe^2+^ with or without FtrB) were aliquoted into a buffered solution of excess ferrozine and allowed to form the characteristic Fe^2+^-ferrozine complex that absorbs at 562 nm (ε_562_ = 27,900 M^–1^cm^–1^).^28^ Absorbances due to the Fe^2+^-ferrozine complex were converted to Fe^2+^ concentrations and plotted against the corresponding time for quenching, as shown in Figure 13.

As seen in Figure 13, the change in [Fe^2+^] vs time graphs at pH 7.3 and 6.8 trace the autoxidation of Fe^2+^ plots (Figure 13C,D), indicating that wild-type Cu^2+^–FtrB did not exhibit the predicted ferrous oxidase activity under these conditions. This could be due to the much higher stability of Fe^3+^ in alkaline medium (making the concentration of free Fe^2+^ very low) which can be concluded by consulting its Pourbix diagram.^29^ However, at pH 6.5, the slope for the change in [Fe^2+^] vs time plot differs from that of Fe^2+^ autoxidation at that pH (blue dots in Figure 11B), indicating that Cu^2+^–FtrB (wild-type) could act as a ferrous oxidase at this pH. The [Fe^2+^] vs time data at pH 6.5 were plotted as zero-, first-, and second-order integrated rate laws, and the best-fit line was determined. The experimental data and the fit showed the best agreement for a zero-order reaction (with respect to Cu^2+^–FtrB, as Fe^2+^ was present in excess), as shown in Figure 13 D. The slope of this best-fit line (Figure 13 B) was used to deduce the observed zero-order rate constant at pH 6.5 (*k*_obs_ = 2 × 10^–8^ M s^–1^) and compared with the rate of autoxidation at this pH (= 7 × 10^–9^ M s^–1^), revealing that the presence of the enzyme enhances the rate of Fe^2+^ oxidation by 3.5-times at this acidic pH. This observation aligns with whole-cell data, where deletion of any of the *ftrABCD* genes at an acidic pH resulted in a phenotype that could not retain viability under iron starvation conditions. Finally, we performed a similar ferrozine assay on the H121A mutant of FtrB as this mutant showed Cu^2+^ affinity. [Fe^2+^] vs time data for this protein in pH 6.5 is presented in Supplementary Figure 4 along with the autoxidation rate of Fe^2+^, and it shows that despite having the Cu^2+^ binding ability, this mutant was not able to perform the ferrous oxidase activity.

## Materials and Methods

All buffers (HEPES, phosphate buffer) used in this work were of highest purity grade and were pH adjusted to the desired experimental conditions using a pH meter (Fisher). Metal stocks were obtained by making at least 2-fold dilutions to atomic absorption standards (Ricca chemicals). Experimental design and data analysis methods for individual experiments are described in the section below.

### Cloning, Expression, and Purification of FtrB Proteins

A translation of the *ftrB* open reading frame was analyzed by SignalP 5.0 to predict the cleavage site of the Sec-dependent amino terminal signal sequence. The mature secreted protein was predicted to start at residue 45 relative to the first amino acid. Accordingly, the sequence from codon 45 through codon 133, the stop codon, was used to construct the protein expression clone in pET-28a (+)-TEV, resulting in pET28-FtrB. The expression construct was generated by GenScript USA, Inc. An NdeI site was engineered 5′0H of the *ftrB* codon 45 and an Xho1 site was engineered at the 3′—OH after the stop codon. The resulting fragment was then assembled into pET-28a (+)-TEV at the Nde1 and XhoI sites such that the His tag and TEV sites are in frame with the mature *ftrB* coding sequence.

*E. coli* BL21 cells containing the pET28(a)-TEV plasmid for mature *ftrB* were shaken at 37 °C in Terrific broth supplemented with 40 μg/mL kanamycin and was induced when the OD600 reached 1.2 by adding 2 mM isopropyl-β-d-thiogalactoside (IPTG). The cells were harvested after 4 h by centrifugation (10,000 × *g* at 4 °C) for 20 min, and the cell pellet was stored at −80 °C overnight. The following morning, the cells were resuspended in Histidine-Wash (His-wash) buffer (50 mM sodium phosphate, 300 mM sodium chloride, 10 mM imidazole, pH 7.4) (50 mL buffer/Lit of culture) followed by lysing the cells by French pressing (3X). This lysate was then centrifuged (25,000 × *g* for 20 min at 4 °C) and the supernatant preserved for downstream purification. His-tagged FtrB proteins (wild-type, D118A, and H121A mutants) were purified through affinity chromatography by applying the supernatant to the HisPur cobalt column (Thermo Scientific) using a gravity flow method. In short, the column was washed with 3 column volumes of Histidine-recharge buffer followed by equilibrating it with Histidine-wash buffer before applying the crude lysate. The His-tagged protein was eluted from the column by applying 10 column volumes of Histidine-Elution Buffer (50 mM sodium phosphate, 300 mM sodium chloride, and 100 mM imidazole, pH 7.4). This elution fraction containing the His-tagged protein was extensively dialyzed overnight (3×) at 4 °C in His-wash buffer to remove the excess imidazole, and the next morning, 50 μL of TEV and 5 μL of β-marcaptoethanol were added to this to digest the His-tag overnight. The tag-free protein was then applied to an equilibrated HisPur Resin in a gravity flow Cobalt column (Thermo Scientific) where the tag-free FtrB came off with the flow through and the free-tag bound to the column. This tag-free wild-type FtrB crude sample was further purified using a Hi-Load Superdex 75/200 PG column, and protein purity was determined by running 16.5% SDS-PAGE on FtrB fractions. The relative molecular weight (rMW) of the purified wild-type protein was determined by performing analytical gel filtration using KTA pure 25 L FPLC (GE Healthcare) and a Superdex 75 Increase 10/300 GL (GE Healthcare) column with a flow rate of 0.5 mL/min in HEPES-buffered saline (HBS) (10 mM HEPES pH 7.3, 140 mM NaCl). The rMW of FtrB was determined using a semilog linear fit using Gel Filtration Standard (Bio-Rad) using the last four globular proteins γ-globulin, ovalbumin, myoglobin, and vitamin B12 with rMW of 132, 56, 17, and 1.3 kDa, respectively (SI Figure 4, SI Table 2). Protein concentrations were determined using A_280_ on a Biospec NanoDrop instrument (Thermo Scientific).

### Protein Crystallization

For all studies in this work that required the use of apo protein, it was prepared by dialyzing the as-isolated protein in the presence of 5 mM sodium azide to chelate any metals bound during purification and redialyzed sequentially in a buffer containing 10 mM HEPES pH (7.4), 140 mM NaCl, to remove sodium azide. This sample was concentrated to 5 mg/mL in a buffer containing 10 mM HEPES (pH 7.4) and 140 mM NaCl and used in sitting drop vapor diffusion crystallization trials with a protein to crystallization reagent ratio of 1:1. FtrB crystals initially grew in a condition containing 0.2 M sodium acetate, 0.1 M Bis–Tris pH (6.5), 25% (v/v) polyethylene glycol 3350. Optimized crystals were obtained from solutions containing 0.1 M sodium acetate pH (4.6), 20% (v/v) polyethylene glycol 3350 supplemented with 20–100 mM MnCl_2_. These initial FtrB crystals diffracted to 1.8 Å limiting resolution, and analysis of Fo–Fc maps following refinement of all protein atoms revealed two large positive densities that were assigned to Mn^2+^ ions. Final optimized crystals diffracting to 1.3 Å limiting resolution were later obtained with additional supplementation of 20–100 mM CuSO_4_ to the crystallization condition. Diffraction quality crystals grew between 2 and 5 days at room temperature and were subsequently harvested and flash frozen in liquid N_2_, supplemented with 10% glycerol.

### Structure Determination, Refinement, and Analysis

Monochromatic X-ray diffraction data was collected at 1.0 Å wavelength on beamline 22-ID (for FtrB) at the Advanced Photon Source at Argonne National Laboratory through SERCAT (South-East Regional Collaborative Access Team). Diffraction data were integrated, scaled, and reduced using the HKL2000 software package.^31^ The FtrB structure was solved via molecular replacement with an initial model obtained from AlphaFold2 using PHASER, as implemented in Phenix. Initial solutions obtained from molecular replacement were iteratively refined using Phenix and manual building using Coot to arrive at a final model with a refined model (*R*_work_/*R*_free_ of 17.9 and 19.8%) (Table 1). Coordination geometries of Mn were verified by Check my Metal Webserver. Representations of all protein structures and electron density maps were generated using PyMol (www.pymol.org/). Schematic wiring diagrams of protein secondary structure were produced using PDBsum (27). A structure-based similarity search was performed using Foldseek to search the PDB100 database in TM-Align mode.^30−39^

### Structural Analysis and Phylogeny of Cup-II Proteins

Crystallographic structures analyzed in this report were downloaded from www.pdb.org: Azurin (PDB: 1XB6); Rusticyanin (PDB: 1E30); FtrB (PDB: 8VUK); N-terminal of EfeO (PDB: 7WGU). Analysis of these structures was performed by UCSF Chimera [https://www.cgl.ucsf.edu/chimera/]. Sequence-based structural analysis was performed by PROMAL-S3D [http://prodata.swmed.edu/promals3d/]. Pairwise comparison of the protein structures and Q-score calculation was performed by PDBeFold [https://www.ebi.ac.uk/msd-srv/ssm/]. For the phylogenetic analysis, the bacterial FtrB sequence database (47 distinct bacterial complete genomes) was prepared from the KEGG Genome database [https://www.genome.jp/kegg/genome/], and the corresponding phylogenetic analysis was performed by www.phylogeny.fr. The cladogram was constructed by using the software Dedroscope.^40^

### Circular Dichroism (CD)

As mentioned in the Introduction, our groups have recently reported a solid-state structure for wild-type Brucella FtrB at pH 4 which has confirmed the presence of 9 β-sheets in this protein arranged in a fashion that produces the overall cupredoxin fold. No structural data are available for this protein, however, at pH ranges where the FtrABCD system is most functionally efficient (around pH 6.8). We used circular dichroic (CD) spectroscopy to investigate the solution secondary structure of the wild-type protein, the peptide (P), and the two mutants, D118A and H121A, under different pH conditions (pH 7.3 and 6.8) to investigate their solution secondary structures and their variation. All CD experiments were performed using a JASCO J-815 CD spectrometer which was maintained at 25 °C. Spectroscopic data were collected using 1 mm quartz cuvettes in the 190–300 nm range, and averages of 8 consecutive scans were used as raw CD signal. Protein/peptide samples (50–55 μM) were prepared by buffer exchanging in 10 mM phosphate buffer at pH 7.3 or 6.8 for all experiments. Concentration of buffer and proteins was optimized by ensuring that the HT voltage remained below 600 V throughout the scan range. Raw CD data were buffer subtracted and normalized to obtain θ values using a standard formula from previous works.^13,20^
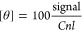
where *C* stands for the concentration of the protein (mM), *n* is the number of amino acid residues on FtrB (without the signal peptide), and *l* is the path length (cm). To obtain CD on wild-type apo-FtrB, the as-isolated protein was first dialyzed against a 5 mM EDTA solution in phosphate buffer, followed by extensive (3×) buffer exchange with phosphate buffer with no EDTA.

Experimental CD data for proteins with predominantly β-sheet secondary structures can be analyzed using the online program BeStSel to determine the % contributions of different secondary structures (α-helix, parallel β-sheets, antiparallel β-sheets, loops, and others). All raw CD data were buffer subtracted and submitted to BeStSel as.txt files (200–250 mm range), and the Single Spectrum Analysis was used to perform the % secondary structure prediction analysis.^17−19^

### Inductively Couple Plasma-Mass Spectrometry (ICP-MS)

ICP-MS was utilized to determine Cu content in the as-isolated wild-type FtrB. As-isolated and apo wild-type FtrB (54 μM) from different preparations in buffer were digested with nitric acid (70%, Fisher, trace metal grade) at 65 °C for 45 min and filtered using a 0.45 μm hydrophilic Teflon filter (SCP Science). This filtered sample was diluted to a final volume of 10 mL using water (Fisher, ultratrace element grade), 1% nitric acid, and spiked with internal standard to a final concentration of 10 μg/L (Inorganic Ventures, IV-ICPMS-71D). An external calibration curve was created using a single element Cu and Mn standard (SCP Science) prepared in 1% nitric acid with a 10 μg/L internal standard and verified by a secondary quality control standard (Inorganic Ventures). Samples were analyzed, and Cu and Mn concentrations were quantified using Inductively Coupled Plasma Mass Spectrometry (Agilent Technologies 7900 ICP-MS, MassHunter 4.2 Workstation Software) operated using the helium collision mode. ICP-MS instrument parameters are shown in SI Table 2.

### Differential Scanning Calorimetry (DSC)

DSC experiments were performed on recombinant wild-type apo-FtrB and wild-type FtrB + 3× Cu^2+^ using a TA Instruments Nano DSC instrument. All experiments were conducted in triplicate to ensure reproducibility. In a typical experiment, the wild-type protein (700 μL, 50–100 μM) was first degassed and then injected into the sample compartment of the DSC instrument. The reference cell was filled with equal volume of buffer solution. Experiments were performed under 3 atmospheric pressures in the 30–100 °C range in a heating–cooling–heating cycle at 2 °C/min scan rate. Raw heat data was buffer subtracted and then converted to molar heat capacity (*C*_p_) vs temperature data using the TA Instruments Nano Analyze program. The *C*_p_ vs temperature data were copied to a Microsoft Excel workbook where Δ*H*, Δ*S*, and Δ*G* over the temperature window were calculated using the following equations.^25^
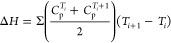

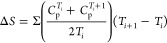

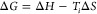


Finally, the Δ*G* versus temperature data were plotted using Microsoft Excel and presented in the Results section.

### Isothermal Titration Calorimetry (ITC)

Samples for ITC were prepared using methods published earlier.^13,25^ In short, wild-type and mutant apo proteins were dialyzed three times in appropriate buffer solutions (100 mM HEPES, 100 mM NaCl (pH 7.3); 50 mM Bis**–**tris, 100 mM NaCl (pH 7.3); 100 mM MES, 100 mM NaCl, pH 7.3). Cu^2+^ and Mn^2+^ solutions were made by diluting atomic absorption standards (Ricca Chemicals) into the third dialysate from apoprotein preparation. All ITC experiments were performed on Nano-ITC (TA Instruments), and raw data were analyzed using Nanoanalyze software. ITC data were analyzed using the following Cu^2+^ or Ag^+^ binding to wild-type apo-FtrB reaction based on the 1:1 binding predicted by the homology model predicted by us.^13,14^
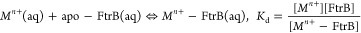
where *M*^*n*+^ = Cu^2+^. [apo-FtrB] (wild-type and H121A mutant) for the Cu^2+^ titration was 100 μM. In a typical experiment, the protein solution was placed in the sample cell of the Nano ITC instrument, and the metal solution was placed in the injector. All experiments were conducted at 25 °C by titrating metal solution incrementally in a well-stirred (320 rpm) protein solution. The raw heat data (after subtracting metal ion into solvent dilution heat) were fitted using an independent model (Nano Analyze, TA Instruments), yielding thermodynamic parameters (*K*_d_, *N*, Δ*H*_ITC_, and Δ*S*).

### Cyclic Voltammetry (CV)

CV experiments were performed on wild-type Cu^2+^–FtrB and Cu^2+^–H121A mutants of FtrB in 100 mM phosphate buffer pH 7.3 using a gold electrode (BASi MF-2114), a Ag/AgCl reference electrode (BASi MF-2052), and a platinum wire counter electrode. The Ag/AgCl reference electrode was calibrated using a [Fe(CN)_6_]^3–^/[Fe(CN)_6_]^2–^ solution following the method published earlier.^41^ Potential sweeps for these experiments were performed using an Epsilon potentiostat connected to a BASi three-electrode system. Self-assembled monolayer (SAM) of the proteins (12 mg/mL) was prepared following the method published earlier.^42^ In short, the gold electrode was first polished and electrochemically acid cleaned to ensure reliability of the Sam formation. The cleaned electrode was then soaked in an ethanolic solution of 10 mM mercaptopropionic acid (MPA) overnight. The next morning, the MPA-modified electrode was gently rinsed with ethanol, followed by air drying. A drop of the respective protein solution was then cast on this electrode and dried under constant N_2_ purging to airdry. The surface adherence of the protein to the electrode surface was confirmed by running a CV experiment with the cleaned bare electrode, the electrode soaked overnight in MPA solution, and electrode with the protein drop cast before each experiment. No redox signal was obtained from the bare gold electrode or gold electrode soaked in MPA in the potential range that was scanned. Before each potential sweep, the buffer solution was purged with N_2_ for ten min followed by blanketing the buffer solution with argon purging during the potential sweep. Supplementary Table 2 provides reduction potential data for characterized cupredoxins and FtrB for the purpose of comparison.

### Ferrous Oxidase Assay

To determine if *Brucella abortus* 2308 FtrB can act as a ferrous oxidase, we utilized a ferrozine assay. This assay has been used in the past for similar purposes and is a robust method to determine the rate of decrease in [Fe^2+^] over time due to enzymes’ ferrous oxidase activity [25]. The success of this assay relies on the ability of ferrozine (Fz) to form a high affinity 3:1 (3 Fz: 1 Fe^2+^) in acidic and physiological pH. This Fe^2+^–Fz complex absorbs at 562 nm and the molar extinction coefficient for this band is known (27,900 M^–1^ cm^–1^).^28^ Reaction mixtures containing known initial concentrations of Fe^2+^ and recombinant wild-type FtrB were quenched at different time intervals with a large excess of ferrozine, and these mixtures were allowed to incubate at room temperature followed by measuring their absorbances at 562 nm. A large excess of ferrozine was used to ensure that there was no free Fe^2+^ in solution that could be oxidized by the protein. As all experiments were conducted in the presence of O_2_, we performed controls under identical conditions on solutions that (a) only contained Fe^2+^ with no wild-type FtrB (autoxidation of Fe^2+^ by O_2_), (b) Fe^2+^ with H121A (H121A mutant binds Cu^2+^), and (c) Fe^2+^ mixed with denatured (heated at 95 °C) recombinant wild-type FtrB (to determine the role of the native protein fold in this proposed oxidation). The raw absorbances were converted to available [Fe^2+^] using the molar extinction coefficient and then plotted against respective time intervals to compare the contribution from autoxidation of Fe^2+^ with the rate of oxidation of Fe^2+^ in the presence of the natively folded wild-type protein. Experiments were conducted with 100 mM HEPES, 100 mM NaCl pH 7.3 and 6.8, and 100 mM Sodium acetate buffer pH 6.5.

In short, the final concentrations of Fe^2+^ and wild-type FtrB in the reaction mixtures were 48 and 15 μM, respectively. Fe^2+^ solutions were made by serial dilution of an atomic absorption standard (Rica Chemicals) in the respective buffers right before the experiments. 200 μL of this solution was aliquoted into 200 μL of Fz solution (final [Fz] = 1 mM) at different time intervals. For the control experiments, everything except natively folded wild-type FtrB was added to the reaction mixture. Finally, absorbance data at different time intervals were converted to residual [Fe^2+^] by dividing it by its reported molar absorptivity values without any volume dilution correction.^28^

### pI and Protein Charge Calculation

The pI and charge on recombinant wild-type FtrB and its mutants were calculated using the ProtPi program (https://www.protpi.ch/) in the pH range 4–8. The one letter amino acid sequences of the proteins were uploaded to this program in FASTA format, and the program computed these values. A plot of pH vs net charge generated by this program is provided as SI Figure 1.

## Conclusions

In this work, we used structural (X-ray diffraction and CD), calorimetric (ITC, DSC), spectroscopic, and electrochemical experiments on recombinant wild-type and two mutant FtrB proteins (D118A and H121A) from *Brucella abortus* 2308 to investigate its structure–function relationship. Both solid-state and solution structure studies confirm β-sheet formation, as predicted by previous evolutionary and homology modeling studies.^14,15^ However, a closer inspection of the solid-state structure and its comparison with characterized cupredoxins show that this FtrB has an additional β-sheet and the H-bond network folding the structure is distinct compared to the structures of known cupredoxins (Figures 5 and 6).^16^ Given that the fast ET reaction performed by classical cupredoxins is modulated by the primary coordination shell of the bound Cu^2+^, number of β-sheets, and the H-bonding network, the finding that *Brucella abortus* 2308 FtrB has unique features in all three aspects can lead to a different mechanism of redox reaction.^16^

We also report multiple sequence analysis of FtrB from various bacterial genomes, revealing the divergence of FtrB evolution based on the presence of FtrP in the open reading frame (Figures 3 and 4). Taking this together with our previous finding that the presence of FtrD in bacterial genomes with either intact *ftrABCD* or *ftrABC* in a single open reading frame also cause divergent evolution of FtrB,^14^ we conclude possible flexibility in FtrB function. Despite this flexibility, all FtrB conserves E86 and E93 residues, which are seen to coordinate to a Fe^2+^ mimic in our crystal structure (Figure 5), and we conclude that FtrB plays a crucial role in iron transport.

The most significant finding in this study is the ability of wild-type *Brucella abortus* 2308 FtrB to bind Cu^2+^ using the conserved Cup-II residues D118 and H121 which were demonstrated by ITC, DSC, and CV data. The absence of the conserved HHC residues from FtrB was a main concern relating to its predicted function as a copper containing ferrous oxidase. Although there is overwhelming evidence for preference of Cu^2+^ to HHC residues in cupredoxins,^16^ previous studies confirm Cu^2+^ binding affinity by mutant azurin where the Cys residue is substituted with an Asp.^43,44^ In addition to these, there are several other studies where proteins with overall cupredoxin folds bind Cu^2+^ in diverse nonclassical sites, such as the His-brace proteins. Taking data from these reports and others, it is crucial we reevaluate the Cu^2+^ sites in biological systems.^45,46^ Finally, electrochemical and spectrophotometric assays demonstrated that wild-type Cu^2+^–FtrB can function as a ferrous oxidase at acidic pH, aligning with the proposed biological role. We determined the pseudozero-order rate constant for Fe^2+^ oxidation by Cu^2+^–FtrB as 2 × 10^–8^ M s^–1^, which was 3.5 faster than the rate of autoxidation of Fe^2+^ at this pH. Interestingly, the D118A and H121A mutants failed to exhibit this activity, underscoring the importance of these Cup-II residues in the enzymatic function of FtrB.

Overall, our findings enhance the understanding of FtrB’s structural dynamics, metal-binding properties, and functional roles, contributing to the broader knowledge of cupredoxin-like proteins and their evolutionary significance.

## Data Availability

The data sets presented in this study can be found in online repositories. The names of the repository/repositories and accession number(s) can be found below: http://www.wwpdb.org/, under the RCSB PDB accession code 8VUK. All other data are contained within the manuscript.

## Supporting Information Available

pH vs net charge for recombinant wild-type FtrA generated by the ProtPi program; structure based sequence alignment of Rusticyanin (PDB: 1E30), Azurin (PDB:1XB6), FtrB (PDB: 8VUK), and N-terminal of EfeO (PDB: 7WGU); bar graph generated from the thermodynamic analysis of Δ*H*_ITC_, Δ*G* (obtainedfrom the *K*_d_), and Δ*S* of Cu^2+^ binding to wild-type and H121A mutant FtrB; [Fe^2+^] vs time data for reaction mixtures with H121A (blue dots) and without (orangedots) in acetate buffer, pH 6.5; size exclusion chromatography and analytical size exclusion chromatography; expression and purification of wild-type FtrB as a monomer; and ^63^Cu concentrations (in ppb) for different samples in 25 mM ACES buffer asobtained from ICP-MS experiments (PDF)

## Abbreviations

CD: circular dichroism
ITC: isothermal titration calorimetry
DSC: differential scanning calorimetry
CV: cyclic voltammetry
ICP-MS: inductively coupled plasma-mass spectrometry
ET: electron transfer
HEPES: 4-(2-hydroxyethyl)piperazine-1-ethanesulfonic acid
ACES: N-(2-acetamido)-2-aminoethanesulfonic acid
