# Supplementary material for: In Vitro Structural and Functional Studies of a Novel Cupredoxin, FtrB, from Brucella abortus 2308
Source: ACS Omega. 2025 Mar 22;10(12):12653–70. doi: 10.1021/acsomega.5c00690 (PMC11966278; doi:10.1021/acsomega.5c00690)
Supplement: Supplementary file 1 — ao5c00690_si_001.pdf [file ao5c00690_si_001.pdf]

SI Material

In Vitro Structural and Functional Studies of a Novel Cupredoxin, FtrB, From *Brucella abortus* 2308

*Alexa Kerkan[1,†] Kai Hart[1,†] Daniel W Martin[2], Jason Pajski[1], Bridget Aidoo[1], Brandon L Garcia[3], Sourav Roy[3], Saumya Dasgupta[4], Shabnam Hematian[5], Andrea Santisteban-Veiga[6,7], Nicholas Joseph Schaaf[1], Sambuddha Banerjee[1]**

[1]*Department of Chemistry, East Carolina University, Science and Technology Building, Room 409, Greenville, NC, USA, 27858,* banerjees17@ecu.edu

[2] *Department of Microbiology and Immunology, Brody School of Medicine, East Carolina University, Greenville, NC, USA, 27852*

[3] *Department of Biochemistry and Molecular Biophysics, Kansas State University, Manhattan, KS, 66506, USA*

[4] *Department of Chemistry, Amity University Kolkata, Kolkata, WB, India, 700134*

[5] *Department of Chemistry and Biochemistry, University of North Carolina at Greensboro, Greensboro, NC 27402, United States*

[6]*AFFINImeter Scientific & Development team, Software 4 Science Developments,*

[7]*University of Santiago de Compostela, Santiago de Compostela 15782, Spain, Colloids and Polymers Physics Group, Institute of Materials (iMATUS), Department of Applied Physics, University of Santiago de Compostela, Santiago de Compostela 15782, Spain*

[†]*These authors contributed equally to this work*

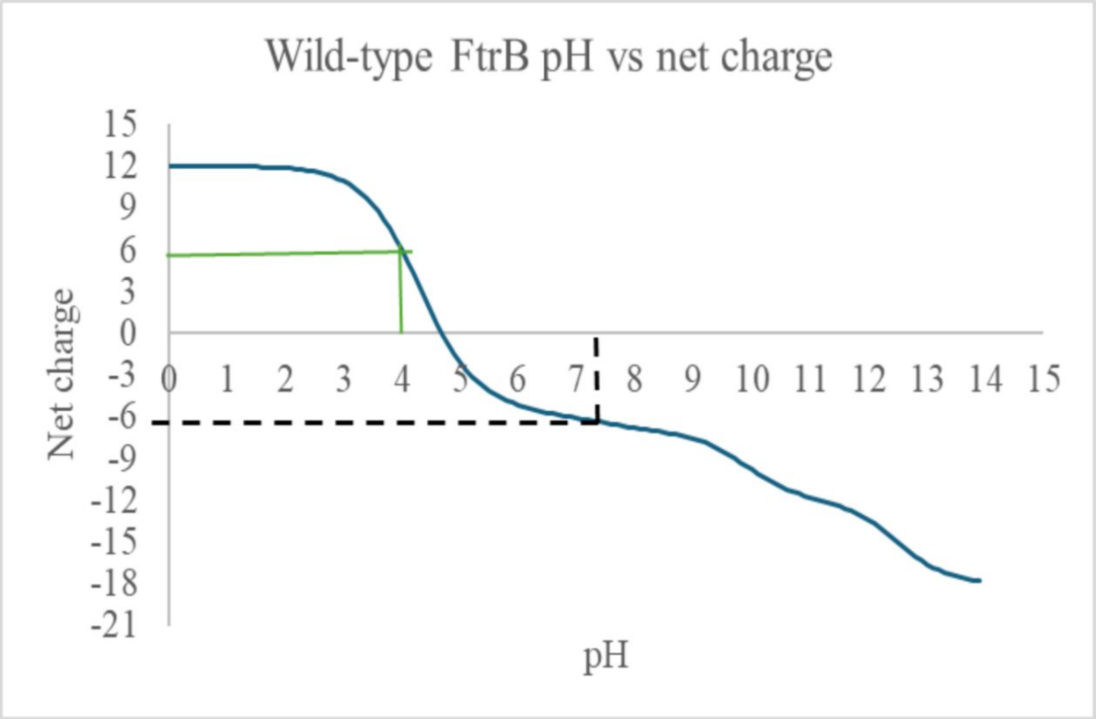

SI Figure 1. The pH vs net charge for recombinant wild-type FtrA was generated by the ProtPi program. The net charge on this protein changes from ~+6 at pH 4 (the crystallization pH) to -6 at pH 7.3. The net charge on the protein at pH 6.5 at which it showed most significant ferrous oxidase activity is -5.88.

β1 **β2**

1e30_chainA_Rustacyanin.p 35 --
LDTTWKEATLPQVKAMLEKDTGKVSGDTVTYSGKTVHVVAAA-
VLPGFPFPSFEVHDKKNPTLEIPA- 100

1xb6_Azurin.pdb_chainA_s0 21 ---------------------------------AECSVDIQGNDQMQFN------------
TNAITVDRS 45

FtrB_refine_52.pdb_chainA 44 ---------------------------------AEEPTFRLEFKD-GVIT------------
PDRLEVPA- 67

N_EfeO.pdb_chainA_s003 1 ---------------------------------ADVPQVKVTVTD-KQCE------------
PMTITVNA- 24

Consensus_aa: ....p*h*c*l*...s.................s..*l*p*l*s..

β3 **β4** **β5**

1e30_chainA_Rustacyanin.p 101 GATVDVTFINTNK----GFG**H**SFDITKK--------------------------
GPPYAVMPVIDPIVA 139

1xb6_Azurin.pdb_chainA_s0 46
CKQFTVNLSHPGNLPKNVMG**H**NWVLSTAADMQGVVTDGMASGLDKDYLKPDDSRVIAHTK---
------- 105

FtrB_refine_52.pdb_chainA 68 NTRFRIELVNTGS-----MPA**E**F**E**SLEL--------------------------
RK**E**KV---------- 95

N_EfeO.pdb_chainA_s003 25 -GKTQFIIQNHSQ-----KAL**E**W**E**ILKGV-----------------------
MVVE**E**RE---------- 54

Consensus_aa: s.p*h*p*h*.*h*.p.sp.....bs*h*p@...p...

β6 **β7** **β8**

1e30_chainA_Rustacyanin.p 140 GTGFSPVPKDGKFGYTNFTWHP----
TAGTYYYV**C**QIPG**H**AATG**Q**FGKIVVK-- 187

1xb6_Azurin.pdb_chainA_s0 106 ---LIGSG----EKDSVTF-DVSKLKEGEQYMFF**C**TFPG**H**-
SAL**M**KGTLTLK-- 148

FtrB_refine_52.pdb_chainA 96 ----IAAQ----SETVMVIRTL----DPGEYPFF**DD**F--**H**-PGG**T**PAILIAK--
132

N_EfeO.pdb_chainA_s003 55 ---NIAPG----FSQKMTANL-----QPGEYDMT**C**GL--L--
TNPKGKLIVKGE 92

Consensus_aa: .....ss..........*hh*.......ps.p**Y**.*hh*s.*h*..**H**..s...t.*lhh***K**..

SI Figure 2. Structure based sequence alignment of Rusticyanin (PDB: 1E30), Azurin (PDB: 1XB6), FtrB (PDB: 8VUK) and N-terminal of EfeO (PDB: 7WGU). Beta strands are highlighted in green α-helices are highlighted in yellow.

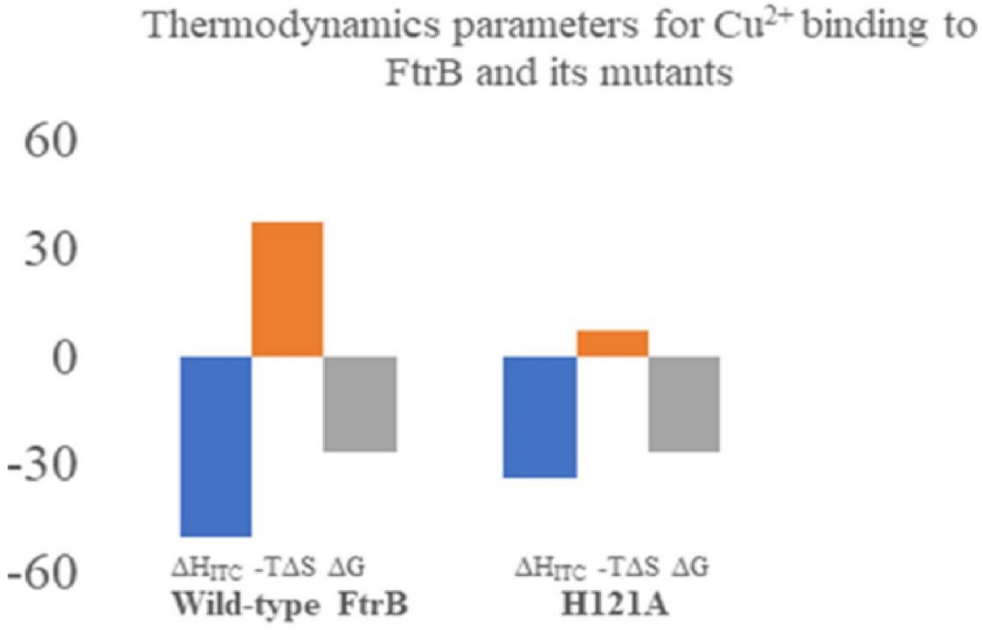

SI Figure 3. Bar graph generated from the thermodynamic analysis of ΔHITC, ΔG (obtained from the K_d), and ΔS of Cu^{2+} binding to wild-type and H121A mutant FtrB. This graph shows ΔH_{ITC} (blue bar), $-T\Delta S$ (orange bar), and ΔG (gray bar). As can be noted, although both the wild-type and H121A mutant showed similar ΔG (based on $\Delta G = RT\ln(1/K_d)$, the ΔH_{ITC} contribution for Cu^{2+} binding to H121A is much smaller and the ΔG has greater contribution from the $-T\Delta S$ term.

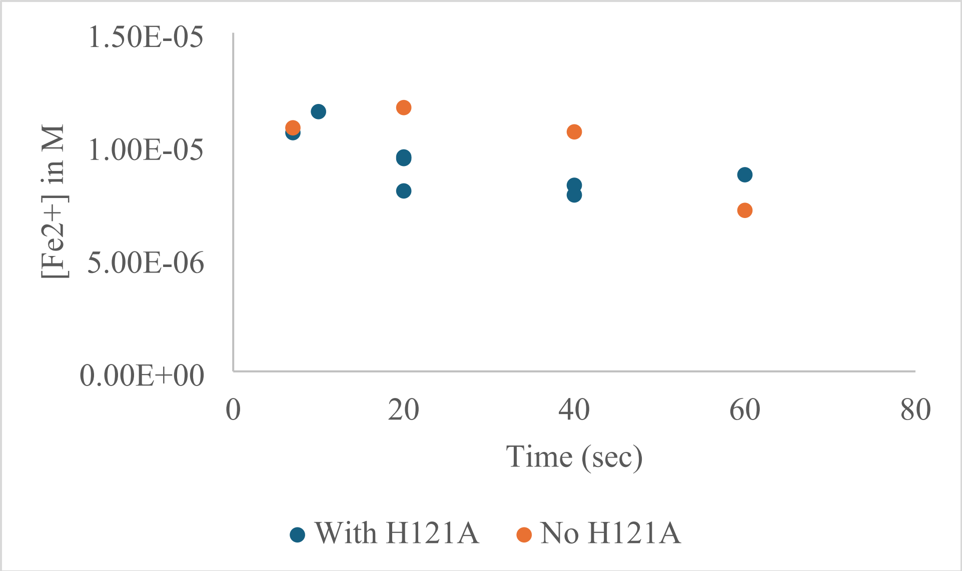

SI Figure 4. [Fe^{2+}] vs time data for reaction mixtures with H121A (blue dots) and without (orange dots) in acetate buffer, pH 6.5.

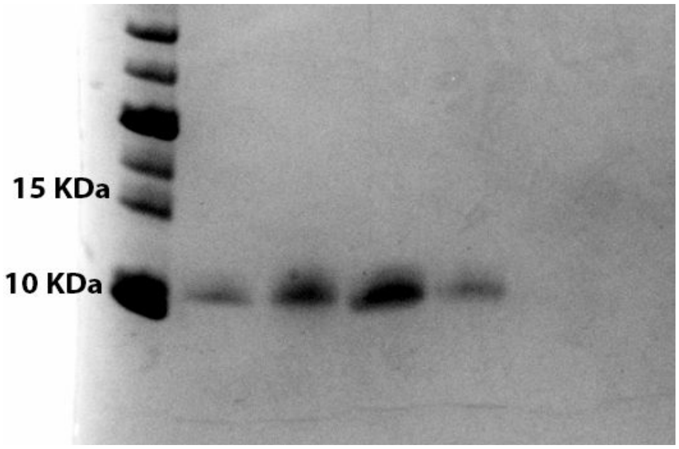

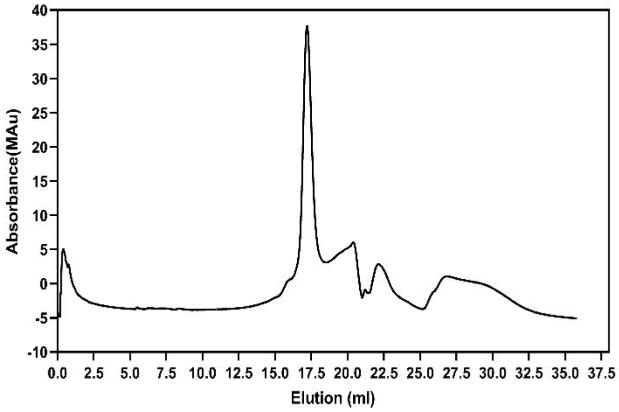

A) B)

SI Figure 5. A) Elution fractions of purified FtrB from size exclusion chromatography on a preparatory grade Hi-Load Superdex 75/200 PG column run on 16% SDS-PAGE. B) Analytical Size exclusion chromatography of FtrB on Superdex 10/300 column with peak elution volume corresponding to a monomeric molecular weight of 14.2 KDa.

SI Table 1. The relative molecular weight of WT FtrB from SEC was found to be 15.3 kDa. FtrB was tested under two different pH conditions and yielded the same relative molecular weight. The theoretical weight of *Brucella* FtrB is 10 kDa. We can conclude from this data that wild type FtrB is expressed and purifies as a monomer.

Protein	R_s (nm)		Da
Ovalbumin	2.8		44000
Myoglobin	1.9		17000
Vitamin B12	0.85		1350
WT FtrB	2.0099	pH 7.3	15343.26

SI Table 2. ^{63}Cu concentrations (in ppb) for different samples in 25 mM ACES buffer as obtained from ICP-MS expreiments.

Sample description				^{63}Cu	[He]
Type	Level	Sample Name	Vial Number	Conc. [ppb]	CPS
Sample		AS Isolate	1112	40.746	806652.39
Sample		AS Isolate spike	1110	138.432	2651530.89
Sample		25 mM ACES Buffer	2303	1.9115	52194.047
Sample		25 mM ACES Buffer spike	2301	104.041	1984855.01
Sample		Blank spike	2105	102.305	1924810.64

SI Table 3.

**FtrB**	Q-score	RMSD	Z-score	Seq-%	**$\%_{sse}$**
Azurin	0.41	2.03	7.0	16	67
Rusticyanin	0.34	2.29	7.1	14	89
N-EfeO	**0.67**	**1.49**	**8.9**	**24**	**100**
